# New QSAR Models to Predict Human Transthyretin Disruption by Per- and Polyfluoroalkyl Substances (PFAS): Development and Application

**DOI:** 10.3390/toxics13070590

**Published:** 2025-07-14

**Authors:** Marco Evangelista, Nicola Chirico, Ester Papa

**Affiliations:** 1 QSAR Research Unit in Environmental Chemistry and Ecotoxicology, Department of Theoretical and Applied Sciences, University of Insubria, via J.H. Dunant 3, 21100 Varese, Italy; mevangelista@uninsubria.it (M.E.); nicola.chirico@uninsubria.it (N.C.); 2 Department of Science and High Technology, University of Insubria, via Valleggio 11, 22100 Como, Italy

**Keywords:** endocrine disruption, human transthyretin disruption, new approach methodologies, PFAS, QSAR

## Abstract

Per- and polyfluoroalkyl substances (PFAS) are of concern because of their potential thyroid hormone system disruption by binding to human transthyretin (hTTR). However, the amount of experimental data is scarce. In this work, new classification and regression QSARs were developed to predict the hTTR disruption based on experimental data measured for 134 PFAS. Bootstrapping, randomization procedures, and external validation were used to check for overfitting, to avoid random correlations, and to evaluate the predictivity of the QSARs, respectively. The best QSARs were characterized by good performances (e.g., training and test accuracies in classification of 0.89 and 0.85, respectively; R^2^, Q^2^_loo_, and Q^2^_F3_ in regression of 0.81, 0.77, and 0.82, respectively) and significantly broader domains compared to the few existing similar models. The application of QSARs application to the OECD List of PFAS allowed for the identification of structural categories of major concern, such as per- and polyfluoroalkyl ether-based, perfluoroalkyl carbonyl, and perfluoroalkane sulfonyl compounds. Forty-nine PFAS showed a stronger binding affinity to hTTR than the natural ligand T4. Uncertainty quantification for each model and prediction further enhanced the reliability assessment of predictions. The implementation of the new QSARs in non-commercial software facilitates their application to support future research efforts and regulatory actions.

## 1. Introduction

Per- and polyfluoroalkyl substances (PFAS) are a large and largely heterogeneous class of human-made compounds, whose strong carbon–fluorine bonds in their structure provide them unique properties (e.g., amphipathic nature, chemical and thermal stability) that have led to their widespread use in different industrial and consumer applications [1]. However, many PFAS and their terminal transformation products are characterized by high persistence in environmental matrices (e.g., surface and groundwater, soils, sediments, atmosphere) due to their resistance to biotic and abiotic degradation processes under environmental conditions [1,2]. Furthermore, some PFAS have been reported to be mobile in the environment, and to bioaccumulate in living organisms [2]. The combination of persistence and mobility of PFAS results in their global contamination [2], leading to increasing exposure of humans and wildlife through multiple pathways, including oral ingestion of contaminated water and food, inhalation of airborne particles, dermal contact with environmental matrices, and consumer and personal care products [3]. Exposure to PFAS has been linked to several serious diseases in mammals, reptiles, fish, birds, and amphibians, including neurotoxicity, immunotoxicity, reproductive impairment, and endocrine disruption [4,5]. The lowest observed effect concentrations (LOECs) of different PFAS have been reported mainly in the ng/mL—µg/mL range in biological matrices, depending on the species and the adverse effect under investigation [5]. PFAS have also been shown to cause toxic effects on terrestrial and aquatic invertebrates, with lethal and effect concentrations to 50% of the population (LC50 and EC50, respectively) mainly reported in the mg/kg or mg/L range [6]. The ubiquitous presence of PFAS and the serious threats they pose raise concerns for human health and the environment that need to be addressed.

Endocrine disruption (ED) may occur through the interference of both legacy and emerging PFAS with multiple molecular targets encompassed in the hypothalamic–pituitary–thyroid (HPT) axis [7]. In mammalian species, the HPT axis is responsible for regulating thyroid hormones (THs) homeostasis [8], the proper functioning of which is critical as THs play a key role in multiple biological functions during both fetal and post-natal life stages [9,10,11,12]. One biological mechanism by which xenobiotics (e.g., PFAS) can interfere with the physiological functions of the HPT axis is the competition with the TH thyroxine (T4) for binding to the human TH distributor protein transthyretin (hTTR), which has been identified as a critical molecular initiating event (MIE) in adverse outcome pathway (AOP) networks for TH system disruption [13,14]. hTTR is involved in a variety of biological functions, including the regulation of abnormal changes in the serum levels of free THs, and the mediation of T4 delivery from blood to cerebrospinal fluid across critical barriers, such as the blood–brain barrier and the placenta, during fetal development [15]. PFAS exposure of vulnerable populations, such as pregnant women, is thus a critical issue [16] as THs regulate brain differentiation and central nervous system formation [17], and play a key role in the metabolism, differentiation, and development of the placenta [18]. Since the embryo/fetus relies entirely on maternal THs during the early stages of gestation, any disruption in THs supply can have significant, even irreversible, consequences that can extend beyond neonatal life [19]. Furthermore, a recent study advanced the hypothesis of the potential multi-transgenerational effects of PFAS on the thyroid axis [20]. For these reasons, the fast identification of substances exhibiting this type of toxicity, defined as thyroid hormone system-disrupting chemicals (THSDCs), is urgently needed [21].

Although associations between PFAS and TH system disruption have been proven, research studies still predominantly target known legacy PFAS. For many others, and in particular for short-chain and emerging variants, such information is heavily limited or absent [22,23,24]. This creates alarming gaps in the understanding of PFAS toxicity and generates substantial challenges in their evaluation on an individual basis [22,23,24]. In this context, the development and application of new approach methodologies (NAMs), including quantitative structure–activity relationship (QSAR) models, is being strongly promoted by authorities [25], intergovernmental organizations [26], and the scientific community [27,28,29] to accelerate the ED assessment of substances, including potential TH system disruption by PFAS [22,23,30,31], and to facilitate data gap filling, prioritization, and grouping strategies [32,33]. To our knowledge, only three studies have so far proposed QSAR models to specifically predict the potential hTTR disruption by PFAS [34,35,36]. However, these studies were affected by several limitations. The first was the use of commercial software, such as HyperChem (Hypercube, Inc., 1115 NW 4th Street, Gainesville, Florida 32601, USA) [37], Dragon (version 5.5 and version 6.0) [38], and alvaDesc [39], to optimize 3D molecular structures and/or calculate molecular descriptors, which may limit the application of these models. In addition, each study used small datasets to develop QSARs, resulting in models with a weak applicability domain (AD), thus limiting the reliability of predictions for a wider range of chemical structures and responses. In particular, the QSARs proposed by Kar et al. [34] and Kovarich et al. [35] were based on a dataset of experimental hTTR binding affinity data measured for 24 PFAS using the radiolabeled [125I]-T4 *in vitro* binding assay (RLBA) [40], which is now not considered as suitable as competitive fluorescence displacement assays and is thus currently not being validated by the European Commission’s European Union Reference Laboratory for alternatives to animal testing (EURL ECVAM) to measure the binding to hTTR [21]. Sosnowska et al. [36] proposed QSARs using a dataset of 44 PFAS for the prediction of the relative potency factor (RPF), calculated as the ratio of the potency of a specific PFAS to the toxic potency of perfluorooctanoic acid. However, the use of perfluorooctanoic acid as the reference compound does not actually reflect the ability of PFAS to compete with T4 for binding to hTTR. In addition, experimental data were measured using the TTR-TRβ CALUX assay. Analogously to the RLBA, this assay is currently not being validated by the EURL ECVAM [21]. Although these QSARs have provided valuable insights, their limitations related to the ADs coverage, the endpoints studied, the dimension of the datasets, the use of proprietary descriptors, and their availability for application emphasize the need for further work in this area. In addition, the availability of multiple models addressing different structural and response domains is strongly encouraged, allowing for the use of the consensus approach to improve the predictive ability of QSARs.

In this work, new classification and regression QSAR models are proposed and applied in a sequential approach with the aim of providing tools for the qualitative and quantitative screening of potential hTTR disruptors. The new models are intended to be applied to first identify hTTR-binding PFAS (by applying the classification QSAR) and then to quantify their T4-hTTR competing potency (by applying the regression QSAR). This work is an innovation compared to our previous regression models for the prediction of hTTR disruption [41], as it is specifically designed to address PFAS. This work introduces significant innovations, as described below, to overcome the limitations affecting the aforementioned QSARs [34,35,36]. The overarching aim is to address current gaps in the field by enhancing the transparency and robustness of QSAR models, and the reliability of QSAR predictions, in order to boost confidence in their use and promote their wider application, as well as to accelerate TH system disruption assessment of a class of priority substances like PFAS. To this end, a newly published dataset [42] was used in this study to generate the models and to provide external validation. The use of this dataset offers four main distinct advantages compared to those modelled in the foreign QSARs. First, it includes experimental hTTR binding affinities consistently measured for 134 heterogeneous PFAS. Its size is about three times larger than the largest considered in the previous studies [34,35,36]. Second, this dataset is sufficiently large to demonstrate the robustness and predictive ability of the QSARs, and particularly to perform rigorous statistical procedures to detect and avoid overfitting and random correlations. It is worth highlighting that no comparable procedures were applied to ascertain that overfitting did not take place in the previous studies [34,35,36]. The size of a modelled dataset influences the statistical validation procedures that should be carried out, according to the Organisation for Economic Co-operation and Development (OECD) principles for QSAR development and validation [43]. Third, unlike existing models, this work uses data homogeneously measured with the 8-anilino-1-naphtalenesulfonic acid (ANSA)-based binding *in vitro* assay [42], which is a fluorescence-based competitive displacement assay that has been identified as a powerful method for identifying potential THSDCs [44] and is currently being validated by the EURL ECVAM to measure the hTTR binding of chemicals [21]. Fourth, the dataset was built by Degitz and colleagues by selecting PFAS by means of a category-based approach to ensure structural diversity [42]. This strategic selection maximized the chemical space coverage within the PFAS family for training predictive models. An additional significant innovation introduced in this study is the quantification of the uncertainty associated with each model and prediction. Generally, whether a QSAR can provide reliable or unreliable predictions is based on the AD defined on the information included in the training set [43]. However, multiple approaches are available to define the AD, which can vary in terms of the constraint degree impacting the reliability of predictions [43]. In this work, beyond the definition of an AD for each model, the uncertainty quantification is introduced to further enhance the reliability assessment of predictions to improve their confidence. Finally, the foreign QSARs relied on proprietary descriptors, which limits their broader application. In contrast, the models proposed in this work rely on descriptors calculated by non-commercial software [45]. Furthermore, the new QSARs have been implemented in the non-commercial software QSAR-ME Profiler beta version 1.02 (freely available for download at the authors’ website https://dunant.dista.uninsubria.it/qsar/). This marks a clear advantage, as the QSARs are made freely available to scientists to aid the assessment of the hTTR disruption by PFAS from their molecular structure, providing not only a clear quantification of their ADs, but also uncertainty in predictions, which is not common in other QSARs. Finally, a case study is proposed to show how the sequential application of classification and regression QSARs can be used to screen large datasets of PFAS, such as the OECD List published in 2018 [46]. This list contains a comprehensive inventory of 4730 PFAS for which the potential hazards are still largely unknown [47].

## 2. Materials and Methods

### 2.1. Modelled Datasets and Data Curation

A dataset was retrieved from a newly published study by the United States Environmental Protection Agency (US EPA) [42], which included hTTR binding affinity values for 134 structurally heterogeneous PFAS. The data were measured using the ANSA-based binding *in vitro* assay, a fluorescence-based competitive displacement assay that has been identified as a powerful methodology for the identification of potential THSDCs [44]. The curation of data led to the exclusion of a total of 11 salts and organometals [48]. In order to develop the classification QSAR, the remaining 123 PFAS were classified as active if the median activity was greater than or equal to 50% (74 PFAS), and as weak/not active if the median activity was smaller than 50% (49 PFAS). Moreover, the active compounds were a priori defined as positive (Class A), while the weak/not active were defined as negative (Class I). Two distinct values of median activity were reported for two PFAS: 14.1% and 10.2% for 1H,1H-Perfluorooctylamine (Chemical Abstracts Service Registration Number, or CASRN, 307-29-9); 93.5% and 93.4% for 1,6-Diiodoperfluorohexane (CASRN 375-80-4). Nevertheless, the presence of these distinct values had no effect on the classification of these compounds. The development of the regression QSAR was based exclusively on PFAS with a quantitative hTTR binding affinity value (quantified in terms of half-maximal effect concentration), which resulted in 68 unique compounds. The modelled endpoint was the logarithm of the relative competitive potency (RP), which is defined as the ratio between the binding affinity of T4 and the binding affinity of a PFAS with hTTR. RP has been used in previous studies to quantify the ability of compounds to compete with T4 for binding to hTTR (i.e., T4-hTTR competing potency) [41,49,50,51,52]. For one compound (1,6-Diiodoperfluorohexane, CASRN 375-80-4), two distinct values of hTTR binding affinity were reported (1.712 µM and 1.848 µM); the arithmetic mean of the corresponding RP values was log-transformed and assigned to the compound of interest. The modelled datasets are reported in Supplementary Materials S1 (Tables S1 and S2).

### 2.2. Calculation of Molecular Descriptors and Dataset Splitting for External Validation

The CASRN of each PFAS was used as input in the US EPA CompTox Chemicals Dashboard [53] to download simplified molecular input line entry system (SMILES) notations, which encode for the molecular structures. To ensure consistency, SMILES notations were canonicalized using Open Babel software v. 2.4.1 [54] and used as input in PaDEL-Descriptor software v. 2.21 [45] for the calculation of fingerprints, one-dimensional and two-dimensional theoretical molecular descriptors. Prior to modelling, an in-house R script (an algorithm previously published by our research group [55]) was used to filter the molecular descriptors in order to reduce useless and/or redundant information. Specifically, descriptors with low variance (i.e., constant value for more than 80% of the compounds), or exhibiting a pairwise correlation > 0.95, or with ranges larger than two orders of magnitude units, were excluded. In order to assess the predictive ability of the models on PFAS not used to train the models, each dataset was split into a training set for QSAR development and a test set for its external validation.

As the literature dataset [42] was built by selecting PFAS through a category-based approach to ensure broad structural diversity within the PFAS family, the splitting “by structure” procedure, already suggested in another study [56], was used to keep this structural diversity across both the training and test sets. This procedure first involved conducting a principal component analysis (PCA) [57] on the dataset using the molecular descriptors as input variables. Then, PFAS were ranked according to their scores along the first component. Based on this ranking, two-thirds of the PFAS were assigned to the training sets, and the remaining one-third was assigned to the test sets. Regarding the dataset used for classification, the splitting procedure was performed independently for each activity class. Finally, the molecular descriptors of the compounds included in the training sets were filtered to exclude redundant and useless information [55].

### 2.3. QSAR Models Development

#### 2.3.1. Classification-Based QSARs

Linear discriminant analysis (LDA) was used as the modelling algorithm. The variable subset selection was performed by applying the step-up procedure previously proposed by Rücker and colleagues [58]. This procedure was applied by means of an in-house developed R script tailored to perform this task [55]. The step-up procedure is described as a sort of stepwise selection [58], and it was chosen over other methods of variable selection (e.g., genetic algorithms) because it allows for the calculation of nested bootstrapped cross-validation in a smaller computational time. This method resulted in populations of the best LDA models ranked by their misclassification rate (MR), which is defined as the percentage of incorrect predictions (i.e., false positives (FPs) and false negatives (FNs) out of the total number of predictions). A linear scoring equation is provided for each class (i.e., Class A and Class I). A compound is assigned to the class associated with the equation that returns the higher score. The overfitting of the variable selection procedure [59] was checked by means of the leave-one-out bootstrap method [60] and evaluated in terms of bootstrapped MR (i.e., MR_BOOTSTRAP_). The flattening or the increase of the MR_BOOTSTRAP_, for an increasing number of modelling descriptors, is indicative of possible overfitting. The quality of the selected models and their predictive ability were evaluated using the following metrics: MR and accuracy (ACC), sensitivity (SN), specificity (SP), and precision (P). The analysis was further supported by the receiver operating characteristic (ROC) curve and area under the curve (AUC). Moreover, in order to minimize the possibility of developing models with coincidental relationships between the response and the descriptors, the probability of coincidental relationship was estimated by performing the step-up procedure for variable subset selection 100 times, using randomized descriptors [58] within their range of values, both considering the nature of the descriptors (i.e., discrete, continuous, binary) or not [55]. Additional details regarding the step-up algorithm and the formulae used to calculate the classification metrics listed above are provided in Supplementary Materials S2.

#### 2.3.2. Regression-Based QSARs

Multiple linear regression (MLR) by means of ordinary least squares (OLS) was used as the modelling algorithm. The variable subset selection was performed by selecting the best combinations of modelling variables using the step-up procedure as in Section 2.3.1. In this case, the step-up procedure resulted in populations of the best MLR models ranked by their coefficient of determination (R^2^). The overfitting of the variable selection procedure [59] was checked by means of the leave-one-out bootstrap, as outlined in Section 2.3.1, and evaluated in terms of bootstrapped mean absolute error (i.e., MAE_BOOTSTRAP_). The probability of coincidental relationships between the molecular descriptors and the response was determined as outlined in Section 2.3.1. The evaluation of the models’ fitting and internal robustness was conducted by using several metrics, i.e., R^2^, MAE, and leave-one-out cross-validated R^2^ (Q^2^_LOO_). The calculations of these metrics are reported in Supplementary Materials S2. Furthermore, the Y-scrambling procedure (50 iterations) was carried out to evaluate chance correlation between the descriptors and the response of the selected model, in terms of the average of R^2^ (R^2^_YS_). Low R^2^_YS_ values are observed in the absence of chance correlation among the model descriptors and the response. Finally, the plot of the residuals was generated to graphically verify the homoscedasticity of the residuals in prediction. As described in Section 2.3.1, the overall procedure was performed using an in-house developed script [55].

#### 2.3.3. External Validation

The predictive ability of the optimal classification and regression models was evaluated on the external test sets identified through the splitting procedure described in Section 2.2. It is important to highlight that the external test sets were not used for the training of the classification and the regression models. Rather, the external validation using the external test sets was performed after the selection of the best models on the basis of the assessment of their fitting and robustness quantified on the training set chemicals. The external predictivity of the selected models was quantified on the basis of predictions generated for the test set. Regarding the classification model, the external predictivity was quantified using ACC, SN, SP, and P, with the support of the ROC curve and AUC. Regarding the regression model, the external predictivity was quantified using the MAE and the external Q^2^_F3_ [61].

### 2.4. Applicability Domains

#### 2.4.1. LDA-QSARs Applicability Domain

The AD of LDA-QSAR models was defined in terms of chemical structure and of post probabilities of the classification event. Specifically, a chemical structure was considered as an outlier if its distance, measured as the average of the three nearest cos α neighbors [62,63], was smaller than the 0.95 quantile of the k-nearest neighbors within the distribution of all training set distances [64]. The endpoint domain [62] was defined by post probability thresholds, which were selected on an arbitrary basis (i.e., for external predictions, the classification was considered uncertain if the post probability fell between 0.25 and 0.75). Following the application of the model, the reliability of each prediction was subjected to further evaluation by comparing its uncertainty, as estimated by Shannon entropy, with the maximum uncertainty calculated for the training set. Predictions with uncertainties within the maximum uncertainty calculated for the training set were considered reliable. Further details on the calculation of Shannon entropy values are provided in Supplementary Materials S2.

#### 2.4.2. MLR-QSARs Applicability Domain

The AD of MLR-QSAR models was quantified using the leverage approach, with the graphical support of the Williams plot for the identification of structural and/or response outliers [43]. The leverage values (i.e., the hat matrix diagonal elements, defined in Supplementary Materials S2) of the compounds, which are a measure of their distance from the centroid of the model, were plotted on the x-axis of the Williams plot. The cut-off value h* is defined as 3 × (*p* + 1)/*n*, where *p* is the number of model descriptors and *n* is the number of compounds included in the training set. The leverage value is indicative of the influence of a compound on the model and the reliability of its prediction. Compounds with leverage values exceeding h* were considered to be structural outliers (predictions become less reliable as the leverage distance increases). The standardized residuals, plotted on the y-axis of the Williams plot, are a measure of the response AD. Compounds with a standardized residual exceeding ±2.5 standard deviation units were considered as response outliers. After the application of the models, the reliability of each prediction was subject to further assessment by comparing their uncertainty (i.e., prediction interval) with the maximum uncertainty calculated for the training set, as well as by comparing the predicted values with the experimental range of the response in the training set. Reliable predictions have uncertainties within the maximum uncertainty calculated for the training set and predicted values within the experimental range of the response in the training set. Details on the calculation of prediction intervals are provided in Supplementary Materials S2.

### 2.5. OECD List of PFAS

The OECD List of PFAS, published in 2018 [46], was used in this work as the basis of a case study to demonstrate the application of the here-proposed LDA-QSAR and MLR-QSAR models to a substantial set of PFAS. This OECD List is an update of a previous data collection by the OECD, published in 2007 [65], to which seventeen publicly accessible information sources were added, and data curation was performed to include only substances with a defined CASRN [46]. Consequently, the OECD List used in this study [46] originally consisted of 4730 different PFAS (in terms of structures, applications, and regulatory status). Prior to the application of the QSARs, the OECD List underwent further data curation to remove polymers, mixtures, salts, organometals, and charged structures, as these chemicals are unsuitable for the application of the proposed models. Furthermore, compounds with ambiguous chemical identifiers, as well as those already included in the modelled datasets, were excluded. Stereoisomers were considered as duplicates in their non-chiral form, since QSAR models generated from simple bidimensional structures do not accurately reflect the spatial conformation of those compounds due to missing information on stereochemistry (for further information, see Table S3 in Supplementary Materials S1). The CASRNs of the remaining PFAS were used as input in the US EPA CompTox Chemicals Dashboard [53] to download the SMILES notation of their structures. The SMILES notations were subsequently canonicalized with Open Babel software v. 2.4.1 [54]. No SMILES notations were available for 62 compounds. The overall curation procedure led to a final dataset consisting of 2934 different neutral organic PFAS, including 53 distinct non-chiral forms of 109 stereoisomers that were initially included in the OECD List. The final dataset is reported in Supplementary Materials S1 (Table S4). The LDA-QSAR model was initially applied to classify PFAS in the curated OECD List as active or weak/not active. Compounds that fell outside the AD of the model, as described in Section 2.4.1, were removed. Subsequently, the MLR-QSAR model was applied to predict the T4-hTTR competing potencies (expressed as Log RP, as described in Section 2.1.) of PFAS identified as active by the LDA-QSAR. As described in Section 2.4.2, compounds that fell outside the AD of the model were removed prior to the analysis of the results. The value Log RP ≥ −1.26, suggested in the literature [52], was used as the threshold to identify strong hTTR binders among those PFAS screened as active by the LDA model.

## 3. Results and Discussion

### 3.1. LDA-QSAR

PCA [57] was performed to study the structural space of the full dataset composed of 123 PFAS prior to modelling. Nine halogenated PFAS (where halogen atoms are intended to be bromine or iodine) were identified as structurally dissimilar to the remaining compounds. In a preliminary modelling attempt using the full dataset, two out of these nine compounds, i.e., heptafluorobutyl iodide (CASRN 374-98-1) and 1,6-dibromododecafluorohexane (CASRN 918-22-9), which is the only brominated PFAS in the dataset, were repeatedly misclassified. It was verified that these outliers had a significant impact on the performances of the models, and they were consequently removed from the dataset.

A new population of LDA-QSARs was developed on the training set chemicals, strictly following the procedure described in Section 2.3.1. The splitting of the original dataset, after removal of the outliers, resulted in 82 chemicals in the training set and 39 in the test set. The best LDA-QSAR model was chosen from a population of the best 25 developed using four variables. This selected number of variables was justified on the basis of a flattening of the MR_BOOTSTRAP_ value, as detected by the bootstrap procedure for models with five, and up to ten, variables (see Figure S1 in Supplementary Materials S2). The performance of the best model, summarized in Table 1, was indicative of the good performance of the LDA-QSAR in terms of fitting and robustness, and considering its external predictive ability when it was applied to the respective test set.

Specifically, the AUC values quantified for the training and the test sets were both 0.85, and the global accuracy and sensitivity were close to 0.90 in both the training and the test sets. The specificity was slightly lower, but remained consistently above 0.80. Furthermore, the probability of coincidental relationship between the molecular descriptors and the response using randomized descriptors, reported in Table 1, was close to zero, thereby providing additional support for the quality of the model. To ensure transparency, the linear scoring equations of the split LDA-QSAR are reported in Supplementary Materials S2 (Equations (S1) and (S2)), along with the ROC plots of the model (Figures S2 and S3). The analysis of the AD (Figure S5) highlighted that the majority of the compounds fell within the AD of the model. However, in the training set, 1,6-diiodoperfluorohexane (CASRN 375-80-4) had a cos α value (0.7690) that deviated considerably from the threshold (cos α (t-95% = 0.9661)), while two other compounds (perfluorohexanoic acid, CASRN 307-24-4; 3,3-bis(trifluoromethyl)-2-propenoic acid, CASRN 1763-28-6) had cos α values (0.9372 and 0.9593, respectively) only slightly lower than the threshold. These results indicate the dissimilarity of these PFAS from the rest of the compounds within the structural space defined by the molecular descriptors selected in the model (i.e., GATS3e, ATSC6p, GATS8m, and MIC2, commented on below). In particular, 1,6-diiodoperfluorohexane was the compound with the lowest value of ATSC6p, and the second highest value of MIC2. Similarly, perfluorohexanoic acid was the compound with the highest value of GATS8m. Finally, 3,3-bis(trifluoromethyl)-2-propenoic acid was characterized by the largest value of GATS3e and by a relatively high value of ATSC6p in comparison with the other compounds. Nevertheless, the model accurately predicted 1,6-diiodoperfluorohexane and perfluorohexanoic acid.

Three molecular descriptors out of the four selected in the model are autocorrelation descriptors, i.e., GATS3e, ATSC6p, and GATS8m. Autocorrelation descriptors represent a vast class of global 2D descriptors that have been extensively used for the development of QSAR models across diverse areas of research [66,67,68]. GATS3e and GATS8m are calculated from the Geary’s autocorrelation coefficient and encode for the spatial distribution along the molecular structure of, respectively, electronegativity at lag 3, and atomic mass at lag 8 [69]. It is interesting to note that classification QSAR models developed in previous studies identified similar autocorrelation descriptors weighted by atomic masses (i.e., GATS3m, HATS6m) as relevant to discriminate between the hTTR binding activity degree of PFAS [35,36]. ATSC6p is calculated from Moreau–Broto’s autocorrelation coefficient [70], and reflects the spatial distribution of polarizability at lag 6 [69]. Interestingly, a recent molecular docking analysis identified hydrogen bonds and hydrophobic interactions as the driving forces of the hTTR binding of PFAS [30], where electronegativity and polarizability are critical factors for the formation of these interactions, respectively. These findings supported the selection of descriptors, such as GATS3e and ATSC6p, which encode these types of electronic properties. Furthermore, the use of autocorrelation descriptors may provide information regarding the length and the configuration of molecular structures as they use topological distances to represent the distances between atoms. The descriptor MIC2 is defined as the modified information content index of the neighborhood symmetry of 2-order, and belongs to the information content descriptor class [69]. Information content descriptors measure the degree of diversity within a molecule and therefore are used to describe its complexity. As described by King and colleagues [71], the MIC indices weigh the individual terms of the information content by the atomic weight of the constituent atoms of a compound. Hence, the resulting values encode for the molecular complexity by taking into account the atomic weight of the constituent atoms. Previous literature work identified the descriptor IC3 (i.e., the information content index of neighborhood symmetry of 3-order, similar to MIC2) as relevant to predict the hTTR binding of PFAS [34]. The selected molecular descriptors are comprehensively described in Supplementary Materials S2 (Table S16).

Only a limited number of compounds were misclassified (nine compounds in the training set and five compounds in the test set), thereby demonstrating the predictive ability of the model. Potential sources of misclassification are discussed as follows. The results of the PCA performed on the molecular descriptors of the model (see Table S5 in Supplementary Materials S1) suggested that the misclassified compounds assigned a priori to a specific class actually fell within the structural space dominated by compounds associated with the other class. The PCA did not reveal any other relevant structural patterns or clusters of misclassified compounds. Therefore, the calculation of similarity values using the Euclidean distance on the molecular descriptors of the model was performed (see Table S6 in Supplementary Materials S1), which confirmed the PCA results. The errors were therefore attributed to a high structural similarity of the misclassified compounds with those belonging a priori to the opposite class, which were instead correctly classified. One possible explanation for these errors is that the molecular descriptors selected in the model are not sufficiently sensitive to small variations in the molecular structure of similar compounds that belong to opposite a priori classes. In other cases, compounds were misclassified due to their post probability values being close to 0.5 (e.g., (perfluorobutyryl)-2-thenoylmethane, CASRN 559-94-4; perfluorobutanoic acid, CASRN 375-22-4; 1H,1H,9H-Perfluorononyl acrylate, CASRN 4180-26-1), or due to their median activity values being close to 50% (e.g., octafluoroadipamide, CASRN 355-66-8; 1H,1H,9H-Perfluorononyl acrylate, CASRN 4180-26-1). Another possible explanation for misclassifications could be the presence of mistakes in the experimental measures used to generate the models. Nevertheless, the proposed LDA-QSAR was likely to be precautionary, which is a favorable attribute in QSARs developed for hazards predictions, since the sensitivity quantified for the training and the test sets were both greater than specificity, and a greater proportion of PFAS were misclassified as active than those misclassified as weak/not active. Furthermore, the post probabilities of PFAS misclassified as weak/not active were found to be closer to 0.5 than those of PFAS misclassified as active.

After the external validation of the model, the training and test sets were pooled together and the entire dataset was used to recalculate the model, thereby capturing all the experimental and structural information included in the full dataset, composed of 121 compounds. The linear scoring equations of the full LDA-QSAR for the two classes are reported below:

(1) Class A score=−47+37×GATS3e+79×ATSC6p+10×GATS8m+39×MIC2+log(0.61)

(2) Class I score=−38+30×GATS3e+73×ATSC6p+8.1×GATS8m+32×MIC2+log(0.39)

(n° Training set = 121; ACC = 0.87; MR = 0.13; SN = 0.95; SP = 0.74; P = 0.85; AUC = 0.85)

The here-proposed models should be applied to discriminate between active and weak/not active compounds belonging to the PFAS group. The Shannon entropy values are reported in Supplementary Materials S1 (Table S7). The ROC plot and the AD plot regarding the LDA-QSAR developed on the full dataset are reported in Supplementary Materials S2 (Figures S4 and S6, respectively).

### 3.2. MLR-QSAR

A preliminary modelling attempt was performed using all 68 compounds included in the regression dataset. This showed the presence of five outliers, which adversely impacted QSARs performances: perfluorooctanesulfonyl fluoride (CASRN 307-35-7), 6:1 fluorotelomer alcohol (CASRN 375-82-6), 1H,1H,8H,8H-perfluoro-3,6-dioxaoctane-1,8-diol (CASRN 129301-42-4), 4H-perfluorobutanoic acid (CASRN 679-12-9), and perfluoro-1,4-diiodobutane (CASRN 375-50-8). Perfluorooctanesulfonyl fluoride is the only sulfonyl fluoride included in the dataset, whose Log RP value is −2.5. This value is considerably lower than the Log RP values for the six compounds in the dataset that are most similar to perfluorooctanesulfonyl fluoride, as identified using PCA (i.e., min Log RP = −0.93; max Log RP = −0.032; mean Log RP = −0.38; median Log RP = −0.35). Similarly, 6:1 fluorotelomer alcohol has the lowest Log RP value (equal to −2.7) among all the other fluorotelomers included in the dataset (i.e., min Log RP = −1.98; max Log RP = −1; mean Log RP = −1.5; median Log RP = −1.5). In a similar manner, 1H,1H,8H,8H-perfluoro-3,6-dioxaoctane-1,8-diol is the compound with the lowest Log RP value (equal to −2.8) among all the per- or polyfluoroethers included in the dataset (i.e., min Log RP = −2.3; max Log RP = −0.0082; mean Log RP = −0.90; median Log RP = −0.69). Comparable results were obtained for compounds with at least one ether bond included in the structure, regardless of the fluorination degree of the carbon atoms involved in that bond (i.e., min Log RP = −2.3; max Log RP = −0.0082; mean Log RP = −0.94; median Log RP = −0.68).

The five mentioned outliers were removed from the dataset. The subsequent splitting procedure resulted in 43 chemicals in the training set and 20 chemicals in the test set, and a new population of MLR-QSARs was developed on the training set chemicals, following the procedure described in Section 2.3.2. The best MLR-QSAR model was chosen from a population of the best 25 developed using three variables. Specifically, the MAE was quantified for different populations of bootstrapped models, at increasing levels of complexity, ranging from one up to six variables. The results of this analysis indicated a progressive increase in MAE_BOOTSTRAP_ values in models with more than three variables (see Figure S7 in the Supplementary Materials S2). The fitting and the robustness of the models, which was checked by leave-one-out cross-validation and randomization of the descriptors, were evaluated using several metrics. These metrics, with the additional support of regression diagnostic plots, were then used to select the optimal QSAR among the population of available models with up to three molecular descriptors (25 models for each variable size). The equation of this best split model based on three molecular descriptors (i.e., piPC5, GGI9, and AATSC0e, commented on below) and the plot of experimental versus predicted response values are reported in the Supplementary Materials (see Supplementary Material S2—Equation (S3), and Figure S8), while the values of the statistical metrics are summarized in Table 2. The values of R^2^ and Q^2^_LOO_, which were around 0.80, confirmed the good performance of the MLR-QSAR in terms of its ability to fit the data and of its internal robustness. Moreover, the absence of chance correlation was confirmed by the probability of coincidental relationships and an R^2^_YS_ close to zero. The calculation of small and consistent values of MAE, both for the training and for the external test set, demonstrated the internal and external predictivity of the model. These results were consistent with the values of R^2^ and Q^2^_F3_, quantified for the test set, which were close to or above 0.80, respectively. Finally, the ratio of the number of training set data to the number of molecular descriptors was 14.3, which significantly exceeded the minimum threshold of 5 that is commonly used to control the risk of chance correlations in a QSAR model [43].

An investigation of the regression diagnostic plots (reported in Supplementary Materials S2) did not highlight any relevant anomalies. All the data points were regularly distributed along the diagonal of the experimental versus predicted response values (Figure S8), while the plot of the residuals confirmed the homoscedasticity of the residuals along the range of the predicted values (Figure S10). The Williams plot (Figure S12) showed, on the y-axis, that the standardized residuals of all the predictions fell within ±2.5 standard deviation units, indicating accurate Log RP predictions. On the x-axis, only two compounds (perfluorobutanoic acid, CASRN 375-22-4; perfluorotetradecanoic acid, CASRN 376-06-7) had leverage values (h = 0.2996, and h = 0.3732, respectively) slightly larger than the cut-off h* (0.2791), which underlined their distance from the centroid of the model, defined by the molecular descriptors selected in the model. Indeed, perfluorobutanoic acid and perfluorotetradecanoic acid were the two compounds with the lowest and the greatest values of the descriptors piPC5 and of GGI9, respectively. The most important descriptor selected in the model, according to the related standardized coefficient, was piPC5, which is a topological descriptor defined as a conventional bond order ID number of order 5 that belongs to the path count descriptor group [69]. The positive sign in Equation (S3) suggested that the T4-hTTR competing potency was positively related to conventional bond order. piPC5 provides information about the length, the form [72], and the linear structure of a compound [73]. As indicated by Jia et al. [73], compounds with multiple linear structures exhibit greater values of piPC5, which is linked to higher hydrophobicity. Therefore, the positive sign of piPC5 in Equation (S3) was consistent with the findings of previous molecular docking analysis that identified hydrophobic interactions as drivers for hTTR binding of PFAS [30], which is justified by the hydrophobic nature of the hTTR binding site for T4 [15]. Furthermore, piPC5 values of chemicals in the training set had a strong positive correlation (0.78) with the chain length, which was recognized in previous molecular docking and QSAR studies as an additional driving structural feature for the hTTR binding of PFAS [30,34,35,74]. In a study by Kovarich et al. [35], the average molecular weight (AMW) of PFAS was also identified as a relevant property to discriminate between hTTR binders and non-hTTR binders. In this study, a strong positive correlation (0.82) was observed between piPC5 and AMW, which suggested again the possible role of the molecular dimension in determining the strength of T4-hTTR competitors. The second most significant molecular descriptor, based on its standardized coefficient, was GGI9, which exhibited a negative correlation with Log RP. GGI9 belongs to the family of topological charge descriptors [69,75]. Topological charge indices evaluate the charge transfer between pairs of atoms, and therefore the global charge transfer, in a molecule. GGI9 is defined as the topological charge index of order 9, thus it encodes for the total charge transfer between atoms placed at a topological distance of 9. Topological charge indices are associated with the molecular dipole moment, encoding information about the potential polar interactions that may contribute to chemical behaviors, such as lipophilicity [30,69]. The topological distance encoded by GGI9 provided further information regarding the length and the configuration of molecular structures influencing the binding to hTTR. In this study, the GGI9 values ranged from 0.04 to 0.74 for 21 out of 43 chemicals in the training set, while for the remaining PFAS, the GGI9 value was 0. Interestingly, when chemicals exhibited GGI9 values of 0, the piPC5 values were lower (from 2.3 to 3.8) compared to the piPC5 values of the other chemicals in the training set (which ranged from 3.6 to 4.6). Therefore, GGI9 provided additional information for differentiating the T4-hTTR competing potency of the most active PFAS (i.e., those with larger values of piPC5), depending on their length and configuration. Finally, AATSC0e was the least influential descriptor in Equation (S3) based on its standardized coefficient, and it is inversely correlated with the response. AATSC0e is an averaged, centered, autocorrelation descriptor calculated from Moreau–Broto’s autocorrelation coefficient [70]. It reflects the spatial distribution of electronegativity along the structure of a compound at lag 0. As previously discussed, former molecular docking analysis identified that hydrogen bonds drive hTTR binding of PFAS [30], where electronegativity plays a key role. This finding is consistent with the observation that compounds in the training set with low values of AATSC0e also exhibited large experimental values of Log RPs. The definitions and the correlations between the molecular descriptors selected in the regression model are reported in Supplementary Materials S2 (Tables S17–S19). In conclusion, in order to use all the experimental and structural information included in both the training and the test sets, the MLR-QSAR was recalibrated on the full dataset (63 chemicals in total). The equation of the full model is reported below, together with metrics for the evaluation of the fitting and the robustness of the model:

(3) $$log\;RP=-2.7\;(\pm 1.3)+1.6\;(\pm 0.26)\times piPC5-3.3\;\left(\pm 0.79\right)\times GGI9-11\;(\pm 2.7)\times AATSC0e$$

(n° Training set = 63; R^2^ = 0.80; MAE_TR_ = 0.28; Q^2^_loo_ = 0.77; R^2^_YS_ = 0.049).

Equation (3) is proposed as the MLR-QSAR model to predict the T4-hTTR competing potency (expressed as Log RP) of new PFAS. As expected, the model has analogous coefficients to those observed in Equation (S2), and consistent values of the statistical metrics and applicability domain. The prediction uncertainty values are reported in Supplementary Materials S1 (Table S8), and diagnostic plots regarding the full MLR-QSAR are reported in Supplementary Materials S2 (Figures S9, S11 and S13).

### 3.3. Case Study: Screening the Potential hTTR Disruption of the PFAS Included in the OECD List

In this section, the screening of the hTTR disruption of the PFAS included in the OECD List was addressed as a case study to demonstrate the application of the here-proposed LDA- and MLR-QSARs in a sequential approach. First, the LDA-QSAR was applied to discriminate active from weak/not active compounds. Second, the MLR-QSAR was used to provide a quantitative estimation of the T4-hTTR competing potency (expressed as Log RP) of the active PFAS. In order to facilitate this procedure, and to make it available to scientists interested in the estimation of the hazardous properties of PFAS from the molecular structure, the QSARs proposed in this study were implemented in the QSAR-ME Profiler beta version 1.02, a non-commercial software freely available online (https://dunant.dista.uninsubria.it/qsar/). The models were applied according to the aforementioned approach to screen the 2934 neutral organic PFAS remaining in the OECD List after the data curation procedure explained in Section 2.5. The original names of the structural categories to which the PFAS belong, as provided in the OECD List, were used in this study to support the analysis and are reported from now on between quotation marks (“”).

The LDA-QSAR was initially applied to discriminate between active and weak/non active PFAS. Predictions falling outside the AD of the model, and/or with a post probability lower than 0.75, were deemed unreliable and excluded from further analysis. As is reported in Table 3, nearly 40% of the predictions were reliable. The structural categories “other PFAA precursors and related compoundsperfluoroalkyl ones” and “fluorotelomer-related compounds” were the most and the least covered by the AD of the LDA-QSAR, respectively. A summary of the AD coverage of the LDA-QSAR, focused on each structural category and described by cause, is reported in Table S9. Interestingly, according to these results, the majority of the PFAS were excluded because of post probability falling below the threshold. This was particularly evident for the “fluorotelomer-related compounds” and the “perfluoroalkane sulfonyl compounds”. Nearly one-third of the PFAS were excluded because they were outside of the structural AD. The category “Other PFAA precursors or related compoundssemifluorinated” was the least represented in the training set, given the large percentage of PFAS falling outside the structural AD. The AD coverage of the LDA-QSAR for each studied PFAS is reported in Table S4.

**Figure 1:**
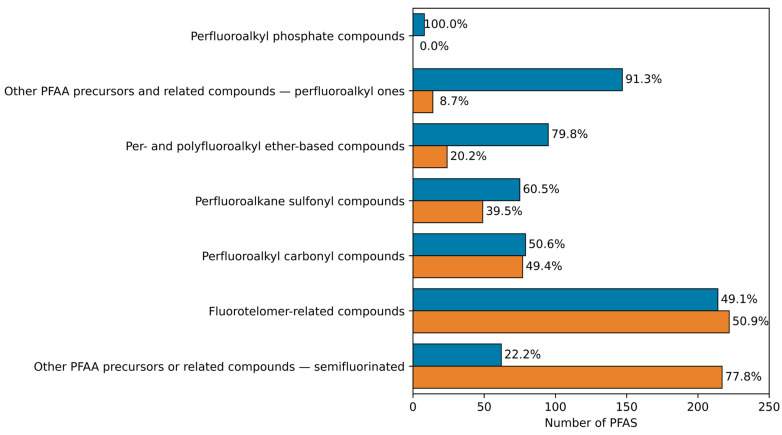
Barplot summarizing the LDA-QSAR screening results for the curated OECD List of PFAS. Blue bars indicate the number of the PFAS predicted as active; orange bars indicate the number of the PFAS predicted as weak/not active. Percentage values indicate the proportion of active or weak/not active PFAS within each structural category.

A total of 680 PFAS (53%) belonging to seven structural categories were predicted as active. As illustrated in Figure 1, the structural categories “perfluoroalkyl phosphate compounds”, “other perfluoroalkyl acids (PFAA) precursors and related compoundsperfluoroalkyl ones”, and “per-and polyfluoroalkyl ether-based compounds” were of major concern due to the high percentage of active predictions. The structural categories “perfluoroalkane sulfonyl”, “perfluoroalkyl carbonyl”, and “fluorotelomer-related compounds”, although showing lower percentages of active predictions, were still of concern given the large number of PFAS belonging to them. Finally, the structural category “other PFAA precursors or related compounds—semifluorinated” was the one of least concern due to the great percentage of weak/not active predictions. Additional details about the proportions of active and weak/not active PFAS, within each structural category and subcategory in which the PFAS were further categorized, as provided in the OECD List, are summarized in Supplementary Materials S1 (Table S10) and more exhaustively described in Supplementary Materials S2. Different properties, encoded by the selected molecular descriptors, were identified as the major drivers of PFAS binding to hTTR (i.e., hydrophobicity, chain length, molecular weight, and electronegativity) and were investigated versus activity profiles across different structural categories. The aim of this analysis was to evaluate whether and how these properties are related to the differences in prediction among various structural categories. The full list of PFAS, along with the abovementioned properties, and a comparative analysis, are reported in Supplementary Materials S1 (Table S11 and Table S12, respectively). As expected, based on the mechanistic interpretation of the selected molecular descriptors, the structural category “other PFAA precursors or related compounds—semifluorinated” was the one characterized by the lowest median values of all the properties under consideration, compared to the other structural categories. On the contrary, the structural categories “perfluoroalkyl phosphate compounds”, “other perfluoroalkyl acids (PFAA) precursors and related compounds—perfluoroalkyl ones”, and “per— and polyfluoroalkyl ether-based compounds” were characterized by high median values of all the properties under consideration, with few exceptions, compared to the other structural categories. Nevertheless, it is important to highlight that the activity is led by the concurrent combination of the molecular descriptors selected in a model.

Following the sequential approach, the MLR-QSAR was then applied to quantitatively predict the T4-hTTR competing potency of the 680 active PFAS. As was mentioned in Section 2.5, the value Log RP ≥ −1.26 suggested in the literature [52] was used as a threshold to identify strong hTTR binders among the active PFAS. As reported in Table 3, after AD assessment, nearly 60% of the predictions were considered reliable. The structural category “other PFAA precursors or related compounds—semifluorinated” was the category least covered by the AD of the MLR-QSAR. With the exception of this structural category, the here-proposed MLR-QSAR was adequately sensitive toward most of the structural features belonging to the different structural categories. A summary of the AD coverage of the MLR-QSAR, focused on each structural category and described by cause, is reported in Table S13, while the AD coverage for each studied PFAS is reported in Table S4.

**Figure 2:**
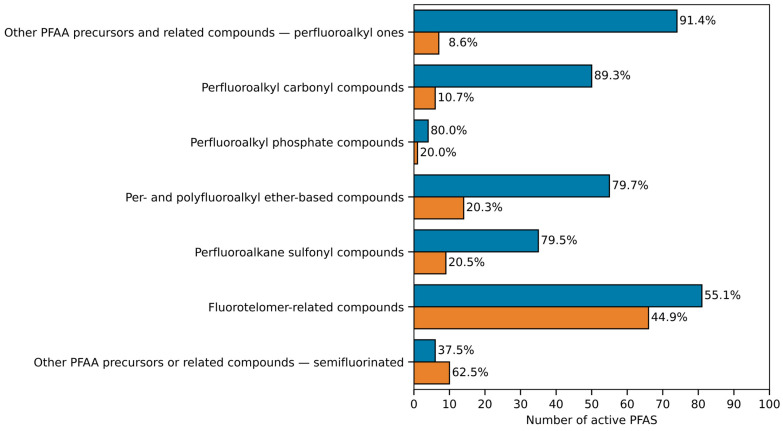
Barplot summarizing the MLR-QSAR screening results for the active PFAS that fell within the AD of the LDA-QSAR. Blue bars indicate the number of the active PFAS predicted with a Log RP ≥ −1.26 (i.e., strong hTTR binders); orange bars indicate the number of the active PFAS predicted with a Log RP < −1.26. Percentage values indicate the proportion of PFAS predicted with a Log RP ≥ −1.26 or <−1.26 within each structural category.

A total of 305 active PFAS (73%) belonging to seven structural categories were predicted as strong hTTR binders. As illustrated in Figure 2, the structural categories “other perfluoroalkyl acids (PFAA) precursors and related compounds—perfluoroalkyl ones”, “perfluoroalkyl carbonyl”, “perfluoroalkyl phosphate compounds”, “per- and polyfluoroalkyl ether-based compounds”, and “perfluoroalkane sulfonyl” were the structural categories of greatest concern due to percentages of strong hTTR binders roughly equal to 80% or more. The structural category “fluorotelomer-related compounds”, although showing a lower proportion of strong hTTR binders, still remained of relative concern due to the large number of PFAS in the category. Finally, the structural category “other PFAA precursors or related compounds—semifluorinated” was the only one predominantly characterized by PFAS of lower hTTR binding strength. Additional details about the proportions of strong hTTR binders among active PFAS, within each structural category and subcategory, are summarized in Supplementary Materials S1 (Table S14) and more exhaustively described in Supplementary Materials S2.

It is also significant to point out that PFAS with positive values of Log RP show a stronger binding affinity to hTTR than its natural ligand T4. Based on results reported in Table S4, a total of 49 PFAS belonging to the structural categories “perfluoroalkyl carbonyl compounds”, “perfluoroalkane sulfonyl compounds”, “per- and polyfluoroalkyl ether-based compounds”, and “other PFAA precursors and related compounds—perfluoroalkyl ones” had positive Log RP values, further indicating a particular need for additional studies on these categories of PFAS.

To provide an additional validation of the predictions generated by the here-proposed QSARs, a thorough and extensive search was conducted for existing experimental data on hTTR disruption within the literature for the PFAS included in the OECD List, among those with reliable predictions. This led to the collection of *in vitro* measured experimental data for only 12 PFAS from six different references [36,40,76,77,78,79]. Four out of these PFAS had multiple data from different studies. The list of the 12 PFAS, along with their experimental outcomes and the corresponding literature references, is reported in Supplementary Materials S2 (Table S20). Nine out of the twelve predictions (i.e., 75%) showed full agreement with all the available experimental data. Remarkably, for three out of these nine PFAS, the predictions fully agreed with multiple data from different studies. These results supported the quality of the here-proposed QSARs. On the contrary, a disagreement between predictions and experimental data was observed for the remaining three PFAS, which were wrongly classified as active. Among these, in one case, the prediction agreed with one of the multiple studies. Nevertheless, it should be highlighted that the predictions were likely to be precautionary. Despite the limited experimental data on hTTR disruption of PFAS, the high level of agreement between them and those generated by the here-proposed QSARs is promising, providing a strong indication of their reliability. Nevertheless, while these results are encouraging, further experimental validation across a broader range of PFAS using new experimental data is desirable.

### 3.4. Comparison with Previous Similar Studies

In the past, a limited number of QSAR studies to predict the potential hTTR disruption of PFAS were performed. However, most of these models used proprietary software during QSARs development, which may limit their application. The models were characterized by narrow structural and response domains because of the small sizes of the training sets, which limited their applicability to a broader range of chemical structures and responses. Furthermore, as was mentioned in the introduction, experimental data used for model development have been measured with inconsistent methodologies (i.e., different assays were used to determine the hTTR binding affinity), and/or assays currently not being validated as reliable and fit for purpose by the European Commission’s EURL ECVAM.

The summary and comparison of the classification and regression QSARs developed in the present work, with those developed in previous studies, are presented in Table 4 and in Table 5, respectively. The presented comparison clearly shows that the new models exhibited similar or lower complexity (i.e., number of descriptors), as well as comparable or better performances than the previous models. Moreover, the new models were based on larger datasets, which included about two to six times the number of chemicals modelled in other studies. Consequently, the new models had larger ADs (see Supplementary Materials S1, Table S15). It is noteworthy that the number of descriptors in the new models was chosen by studying the behavior of MR_BOOTSTRAP_ values in classification and MAE_BOOTSTRAP_ values in regression, with the aim of reducing the risk of overfitting. It is important to note that in the former studies, no comparable procedures were applied to ascertain that overfitting did not take place. Even if the ratio “training set size/number of descriptors” was above or equal to five in all the regression models, which is a simple measure to minimize the risk of chance correlations [43], this did not exclude the possibility of overfitting in the previous QSARs, considering the smaller dimensions of their training sets. Furthermore, unlike the other QSARs, the here-proposed models were not based on commercial descriptors, which enhances their applicability. Nevertheless, the consistency of descriptors across all the different models, even if calculated using different software and selected using different algorithms while modelling different specific endpoints, validated the significance of lipophilicity, chain length, and molecular weight as particularly relevant for the assessment of PFAS activity as hTTR disruptors.

The implementation of the new models into dedicated, and freely available, software facilitated their application for screening purposes, with the clear quantification of their domains and of the uncertainty of predictions. It represented a clear advantage, compared to other models, to assist in the screening of larger numbers of PFAS, such as those included in the OECD List presented in the case study.

## 4. Conclusions

Only a limited number of studies are available that report data on TH activity by PFAS. Consequently, the number of previously published QSARs was limited, and they were based on commercial descriptors, and though characterized by good performances, they only had small ADs. Now, new, simple, robust, and predictive QSAR models were developed in this study to assess the capability of PFAS to bind to hTTR and disrupt hTTR function, which is a critical mode of action known to disrupt the TH system. Two QSAR models were proposed, one to identify hTTR-binding PFAS, and another to quantify their ability to compete with the thyroid hormone T4 for binding to hTTR, in terms of relative competitive potency. The new models were calibrated on larger and homogeneous datasets, including two to six times the amount of data compared to those available for previous models, including most of the chemicals used in previous studies, in addition to tens of other PFAS. Therefore, they had larger ADs and a greater ability to provide reliable predictions for a broader range of PFAS. The size of the dataset allowed for the application of rigorous statistical procedures to detect and avoid overfitting and random correlations, as well as to demonstrate the predictive ability of the QSARs. The statistical metrics calculated for the new models demonstrated their robustness and their capacity to predict the activity of PFAS that had not been used to train the models. In addition, the molecular descriptors, selected in the models by statistical procedures, were consistent with previous *in vitro* and *in silico* findings regarding the major drivers of PFAS binding to hTTR. These findings highlighted the importance of hydrogen bond formation and of hydrophobic interactions, and they pointed out the relevance of lipophilicity, molecular weight, and the chain length of molecular structures. Moreover, similar descriptors were selected in previous QSARs, which were developed using different quantitative approaches and data, thus strengthening confidence in the relevance of these descriptors to describe PFAS binding to hTTR. The utility and the applicability of the QSARs proposed in this study were demonstrated by screening about 3000 compounds included in the OECD List of PFAS. To this end, the models were implemented in the non-commercial software QSAR-ME Profiler beta version 1.02 (freely downloadable at https://dunant.dista.uninsubria.it/qsar/), allowing for the clear quantification of their domains and of the uncertainty of predictions, to further enhance the assessment of their reliability, in order to improve their confidence. The screening allowed for the identification of the PFAS of major concern for their potential hTTR disruption, which were found to belong mainly to the structural categories “per- and polyfluoroalkyl ether-based compounds”, “other PFAA precursors and related compounds—perfluoroalkyl ones”, “perfluoroalkyl carbonyl”, and “perfluoroalkane sulfonyl compounds”. These quantitative results pointed out both categories of PFAS and individual compounds that are of potential concern, suggesting prospects for future research efforts. Though the new models have improved predictive capacity, the screening revealed structural categories that are still poorly covered by the AD of the proposed models (e.g., “other PFAA precursors or related compounds—semifluorinated”) and are thus associated with a high number of unreliable predictions. These findings highlighted the need for additional *in vitro* testing in those areas poorly covered by the AD of the models, with the aim of enhancing the quality and extending the domain of the reliable application of the existing QSARs to a greater number of PFAS. The models and predictions generated in this study addressed a critical gap in the understanding of PFAS toxicity to the functioning of the TH system. As the here-proposed QSARs were developed and validated specifically for PFAS, their application should not be extended to other chemical classes. These findings would support the current general need for NAMs development, and particularly to improve the *in silico* hazard assessment of potentially dangerous chemicals in general, but especially for PFAS due to their environmental and health impacts, and the large number of chemicals in this group. While the new QSARs demonstrated robustness and high predictive performances, which were even confirmed through the validation with experimental data, and they offer valuable simplicity, interpretability, and ease of use, future research efforts could explore the application of more complex machine learning approaches. This could offer new insights and potentially boost the predictive ability in certain structural areas, albeit at the expense of straightforward transparency and simple use. Finally, the implementation of the QSARs proposed in this work into a dedicated and non-commercial software (i.e., QSAR-ME Profiler beta version 1.02) made them available to scientists, industry, and regulatory bodies to facilitate their application and to support the assessment of unstudied and new PFAS, to identify safer alternatives, and to inform future research studies and regulatory actions, particularly for grouping strategies development and prioritization.

## Figures and Tables

**Table 1:** Summary of the statistical results of the LDA-QSAR. “Random range” and “random descriptors nature” indicate the probability of coincidental relationships between the molecular descriptors and the response, using randomized descriptors within their numerical ranges (random range), or considering both their numerical ranges and their nature, i.e., discrete, continuous, binary (random descriptors nature).

	n	ACC	MR	SN	SP	P	AUC	MR_BOOTSTRAP_	Random Range	Random Descriptors Nature	Selected Molecular Descriptors
Training	82	0.89	0.11	0.92	0.84	0.90	0.85	0.32 ± 2.7 × 10^−3^	3.8 × 10^−3^	4.6 × 10^−3^	GATS3e, ATSC6p, GATS8m, MIC2
Test	39	0.85	0.15	0.88	0.80	0.88	0.85	-	-	-	-

**Table 2:** Summary of the statistical results of the MLR-QSAR. “Random range” and “random descriptors nature” indicate the probability of coincidental relationships between the molecular descriptors and the response, using randomized descriptors as defined in Table 1.

	n	R^2^	MAE	Q^2^_loo_	Q^2^_F3_	R^2^_YS_	MAE_BOOTSTRAP_	Random Range	Random Descriptors Nature	Selected Molecular Descriptors
Training	43	0.81	0.30	0.77	-	0.072	0.58 ± 5.7 × 10^−3^	8.3 × 10^−10^	3.3 × 10^−10^	piPC5, GGI9, AATSC0e
Test	20	0.77	0.26	-	0.82	-	-	-	-	-

**Table 3:** The coverage (AD) of the curated OECD List in the LDA-QSAR and in the MLR-QSAR, following the sequential approach, for each structural category.

	LDA-QSAR			MLR-QSAR		
Structure Category	Total (%)	Inside AD		Total (%)	Inside AD	
-	-	Number of PFAS (%)	Number of Structural Subcategories	-	Number of PFAS (%)	Number of Structural Subcategories
Fluorotelomer—related compounds	1086 (37.0)	436 (40.1)	24	214 (31.5)	147 (68.7)	19
Other PFAA precursors or related compounds—semifluorinated	686 (23.4)	279 (40.7)	8	62 (9.1)	16 (25.8)	6
Perfluoroalkyl carbonyl compounds	359 (12.2)	156 (43.5)	9	79 (11.6)	56 (70.9)	6
Per- and polyfluoroalkyl ether-based compounds	280 (9.6)	119 (42.5)	18	95 (14.0)	69 (72.6)	15
Perfluoroalkane sulfonyl compounds	271 (9.2)	124 (45.8)	9	75 (11.0)	44 (58.7)	7
Other PFAA precursors and related compounds—perfluoroalkyl ones	240 (8.2)	161 (67.1)	10	147 (21.6)	81 (55.1)	10
Perfluoroalkyl phosphate compounds	12 (0.4)	8 (66.7)	2	8 (1.2)	5 (62.5)	2
Total	2934 (100)	1283 (43.7)	-	680 (100)	418 (61.5)	-

**Table 4:** Comparative analysis between the present LDA-QSAR and classification QSARs reported in previous research. * Four different models were presented. ** Values of SN, SP, and ACC were calculated in this study from data reported in the Supporting Information of the original paper. kNN: k-nearest neighbor; DTC: decision tree classifier. N/A stands for “not available”.

	Present Model	Kar et al. [34]	Kovarich et al. * [35]	Sosnowska et al. ** [36]
Endpoint	hTTR binding affinity	hTTR binding affinity	hTTR binding affinity	RPF
*In vitro* assay	ANSA-based [42]	RLBA [40]	RLBA [40]	TTR-TRβ CALUX [36]
Method	LDA	LDA	kNN	DTC
Dataset size	121	24	19	44
Training set size	82	16	10	33
Test set size	39	8	9	11
Number of descriptors	4	3	2	2
SN training	0.92	1	0.83–1	0.96
SN test	0.88	1	1	1
SP training	0.84	0.83	0.75–1	1
SP test	0.80	1	0.75–1	0.50
ACC training	0.89	0.94	0.90–1	0.97
ACC test	0.85	1	0.90–1	0.91
P training	0.90	0.91	N/A	1
P test	0.88	1	N/A	0.90
AUC training	0.85	0.95	N/A	N/A
AUC test	0.85	1	N/A	N/A

**Table 5:** Comparative analysis between the present MLR-QSAR and regression QSARs reported in previous research. * Thirty-one different models were reported in Sosnowska et al. [36]; the sizes of the training and test sets were extracted from the Supporting Information of the original paper. IC50: inhibitory concentration 50%. N/A stands for “not available”.

	This Model	Kar et al. [34]	Sosnowska et al. [36] Approach 1	Sosnowska et al. [36] Approach 2 *
Endpoint	RP	IC50	RPF	RPF
Method	MLR	MLR	MLR	MLR
*In vitro* assay	ANSA-based [42]	RLBA [40]	RLBA [40]	TTR-TRβ CALUX [36]
Dataset size	63	15	35	35
Training set size	43	10	24	25
Test set size	20	5	11	10
Number of descriptors	3	2	3	4–5
Ratio training set size/ number of descriptors	14.3	5	8	5–6.3
R^2^	0.81	0.86	0.77	N/A
R^2^_EXT_	0.77	0.64	N/A	N/A
MAE_TR_	0.30	N/A	0.43	N/A
MAE_TEST_	0.26	0.11	0.40	0.34–0.54
Q^2^_loo_	0.77	0.73	0.77	0.76–0.82
Q^2^_F3_	0.82	N/A	0.81	0.76–0.82
R^2^_YS_	0.07	N/A	0.13	N/A

## Data Availability

The original contributions presented in this study are included in the article/Supplementary Materials. Further inquiries can be directed to the corresponding authors.

## Supplementary Materials

The following supporting information can be downloaded at https://www.mdpi.com/article/10.3390/toxics13070590/s1. Supplementary Materials S1, Supplementary Materials S2. Supplementary Materials S1 (Table S1: Dataset for the development of the LDA-QSAR; Table S2: Dataset for the development of the MLR-QSAR; Table S3: Isomeric structures included in the OECD List of PFAS; Table S4: Curated OECD List of PFAS and predictions; Table S5: PCA score plots based on molecular descriptors from the selected LDA-QSAR; Table S6: Euclidean distance-based similarity matrix based on molecular descriptors of the selected LDA-QSAR; Table S7: Shannon entropy values associated to the training set of the full LDA-QSAR; Table S8: Prediction interval (uncertainty) values associated to the training set of the full MLR-QSAR; Table S9: Summary of PFAS removed after LDA-QSAR AD assessment, by cause and by structural category; Table S10: Summary of LDA-QSAR predictions for each structural subcategory; Table S11: List of compounds inside the AD of the LDA-QSAR and their properties; Table S12: Summary table of the information in Table S11; Table S13: Summary of PFAS removed after MLR-QSAR AD assessment, by cause and by structural category; Table S14: Summary of MLR-QSAR predictions for each structural subcategory; Table S15: List of compounds used in this and in previous similar studies, with comparative PCA score plots). Supplementary Materials S2 (description of the step-up procedure; definitions of the statistical metrics for the LDA-QSAR and the MLR-QSAR evaluation; definition of the HAT matrix; uncertainty quantification (i.e., prediction interval and Shannon entropy); Figure S1: Bootstrap analysis of the LDA-QSARs; Equations (S1) and (S2): Linear scoring equations of the split LDA-QSAR; Equation (S3): Equation of the split MLR-QSAR; Figures S2 and S3: ROC curves of the split LDA-QSAR calculated for the training and the test sets, respectively; Figure S4: ROC curve of the full LDA-QSAR; Figures S5 and S6: AD plots for the event A of the split and full LDA-QSARs, respectively; Figure S7: Bootstrap analysis of the MLR-QSARs; Figures S8 and S9: Plots of experimental vs predicted Log RP values of the split and full MLR-QSARs, respectively; Figures S10 and S11: Residuals plots of the split and full MLR-QSARs, respectively; Figures S12 and S13: Williams plots of the split and full MLR-QSARs, respectively; Table S16: Description and definition of selected molecular descriptors in the LDA-QSAR; Table S17: Description and definition of selected molecular descriptor in the MLR-QSAR; Tables S18 and S19: Correlation matrices of selected molecular descriptors in the MLR-QSAR; Table S20: List of PFAS with *in vitro* experimental and predicted outcomes, and literature references; analysis of the predictions of the LDA-QSAR and MLR-QSAR within structural subcategories; references).

## Abbreviations

The following abbreviations are used in this manuscript:

ACC	Accuracy
AD	Applicability domain
AMW	Average molecular weight
ANSA	8-Anilino-1-naphtalenesulfonic acid
AOP	Adverse outcome pathway
AUC	Area under the curve
CASRN	Chemical Abstracts Service Registration Number
DTC	Decision tree classifier
EC50	Effect concentration 50%
ED	Endocrine disruption
EURL ECVAM	European Union Reference Laboratory for Alternatives to Animal Testing
FN	False negative
FP	False positive
HPT	Hypothalamic–pituitary–thyroid
hTTR	Human transthyretin
IC50	Inhibitory concentration 50%
kNN	k-Nearest neighbor
LC50	Lethal concentration 50%
LOEC	Lowest observed effect concentration
LDA	Linear discriminant analysis
MAE	Mean absolute error
MIE	Molecular initiating event
MLR	Multiple linear regression
MR	Misclassification rate
NAMs	New approach methodologies
OECD	Organisation for Economic Co-operation and Development
OLS	Ordinary least squares
P	Precision
PCA	Principal component analysis
PFAS	Per- and polyfluoroalkyl substances
QSAR	Quantitative structure–activity relationship
RLBA	Radiolabeled [125I]-T4 *in vitro* binding assay
ROC	Receiver operating characteristic
RP	Relative competitive potency
RPF	Relative potency factor
SMILES	Simplified molecular input line entry system
SN	Sensitivity
SP	Specificity
T4	Thyroxine
TH	Thyroid hormone
THSDCs	Thyroid hormone system-disrupting chemicals
US EPA	United States Environmental Protection Agency
