# Supplementary material for: New QSAR Models to Predict Human Transthyretin Disruption by Per- and Polyfluoroalkyl Substances (PFAS): Development and Application
Source: Toxics. 2025 Jul 14;13(7):590. doi: 10.3390/toxics13070590 (PMC12300718; doi:10.3390/toxics13070590)
Supplement: Supplementary file 1 [file toxics-13-00590-s001.zip › toxics-3726488_Revised_S2-english done_AfterProofreading.pdf]

Supplementary Materials S2

New QSAR Models to Predict Human Transthyretin Disruption by Per- and Polyfluoroalkyl Substances (PFAS): Development and Application

Marco Evangelista [1,2], Nicola Chirico [1] and Ester Papa [1,*]

[1] QSAR Research Unit in Environmental Chemistry and Ecotoxicology, Department of Theoretical and Applied Sciences, University of Insubria, via J.H. Dunant 3, 21100, Varese (Italy)

[2] Department of Science and High Technology, University of Insubria, via Valleggio 11, 22100 Como (Italy)

Corresponding author:

E-mail address: ester.papa@uninsubria.it

Full postal address: Department of Theoretical and Applied Sciences, University of Insubria, via J.H. Dunant 3, 21100, Varese (Italy)

Table of Contents

- Variable subset selection procedure: step-up 4
- Metrics for the LDA-QSAR evaluation 4
- Metrics for the MLR-QSAR evaluation 6
- Hat matrix (or Leverage Matrix or Influential Matrix) 7
- Uncertainty 7
  - Prediction interval 7
  - Shannon entropy 7
- Bootstrap analysis: LDA-QSAR 8
- Linear scoring equations of the split LDA-QSAR 8
- Equation of the split MLR-QSAR 10
- ROC curves of the split LDA-QSAR calculated for the training and test set, and for the full LDA-QSAR 11
- Applicability domain plots (event A) of the LDA-QSAR (split and full) 14
- Bootstrap analysis: MLR-QSAR 16
- Regression diagnostic plots of the MLR-QSAR: scatterplot of experimental vs predicted Log RP values (split and full) 17
- Regression diagnostic plots of the MLR-QSAR: Residuals plot (split and full) 19

- Regression diagnostic plots of the MLR-QSAR: Williams plot (split and full) 21
- Summary table of the selected molecular descriptors in the LDA-QSAR 23
- Summary table of the selected molecular descriptors in the MLR-QSAR and correlation matrices (training set and full dataset) 24
- Summary table of the predicted PFAS with data from *in vitro* experimental studies........... 26
- Analysis of the predictions of the QSARs within structural subcategories 28

    Analysis of the predictions of the LDA-QSAR within structural subcategories........ 28

    Analysis of the predictions of the MLR-QSAR within structural subcategories 30
- References 32

Variable subset selection procedure: step-up

The step-up procedure [1] was applied to identify the optimal subsets of p molecular descriptors from a large pool. The procedure starts by developing all 1-descriptor models and selecting the best m (in this work, m = 25) in terms of fitting (MR for classification, MAE for regression). To develop 2-descriptor models, one descriptor at a time, selected from the whole pool, is added to each of the previous best m, and their performance is evaluated, still in terms of fitting. As for 1-descriptor models, only the best m ones are selected. The procedure is then repeated up to p molecular descriptors.

Metrics for the LDA-QSAR evaluation

Misclassification rate (MR):

$$MR = \frac{(FP + FN)}{(TP + TN + FP + FN)}$$

Accuracy (ACC):

$$ACC = \frac{(TP + TN)}{(TP + TN + FP + FN)}$$

Sensitivity (SN):

$$SN = \frac{TP}{(TP + FN)}$$

Specificity (SP):

$$SP = \frac{TN}{(TN + FP)}$$

Precision (P):

$$P = \frac{TP}{(TP + FP)}$$

Where

TP: number of true positives;

TN: number of true negatives;

FP: number of false positives;

FN: number of false negatives.

Metrics for the MLR-QSAR evaluation

Coefficient of determination (R^2):

$$R^2 = 1 - \frac{\sum_{i=1}^{n}(y_i - \hat{y}_i)^2}{\sum_{i=1}^{n}(y_i - \bar{y})^2}$$

Mean average error (MAE):

$$MAE = \frac{\sum_{i=1}^{n}|y_i - \hat{y}_i|}{n}$$

Leave-one-out cross-validated R^2 (Q^2_{LOO}):

$$Q^2_{LOO} = 1 - \frac{\sum_{i=1}^{n_{TR}}\left(y_i - \hat{y}_{i/i}\right)^2}{\sum_{i=1}^{n_{TR}}(y_i - \bar{y})^2}$$

External validation metric Q^2_{F3}:

$$Q^2_{F3} = 1 - \frac{\frac{\sum_{i=1}^{n_{TEST}}(y_i - \hat{y}_i)^2}{n_{TEST}}}{\frac{\sum_{i=1}^{n_{TR}}(y_i - \bar{y})^2}{n_{TR}}}$$

Where

$\bar{y}$: average of the experimental responses;

y_i: experimental value of the i-th chemical;

$\hat{y}_i$: predicted value of the i-th chemical;

n: number of compounds in either the training or test set, unless otherwise specified, according to which the metric refers to;

$\hat{y}_{i/i}$: predicted value for the i-th chemical when removed from the model, calculated without the i-th chemical.

Hat matrix (or Leverage Matrix or Influential Matrix)

$$Hat\ matrix = X(X^TX)^{-1}X^T$$

Where

X: data matrix consisting of n rows (i.e., compounds) and p columns (i.e., molecular descriptors).

Uncertainty

Prediction interval:

In regression, prediction uncertainty (i.e., prediction interval) (for further details, see https://dunant.dista.uninsubria.it/qsar/) is calculated as

$$uncert = \pm t_{stud\ \alpha/2} \times s \times \sqrt{1 + HAT_i}$$

Where

$t_{stud\ \alpha/2}$: t-student calculated for α/2 = 0.025,

s : $\sqrt{\frac{\sum_n (y_n - \hat{y}_n)^2}{n-p-1}}$, where n is the number of compounds in the training set and p is the number of molecular descriptors.

Shannon entropy:

In classification, prediction uncertainty is estimated by the Shannon entropy (for further details, see https://dunant.dista.uninsubria.it/qsar/), calculated as

$$Shannon\ entropy = -\sum_{i=1}^{n} p_i\ \log p_i$$

Where

p_i is the event i post probability.

Bootstrap analysis: LDA-QSAR

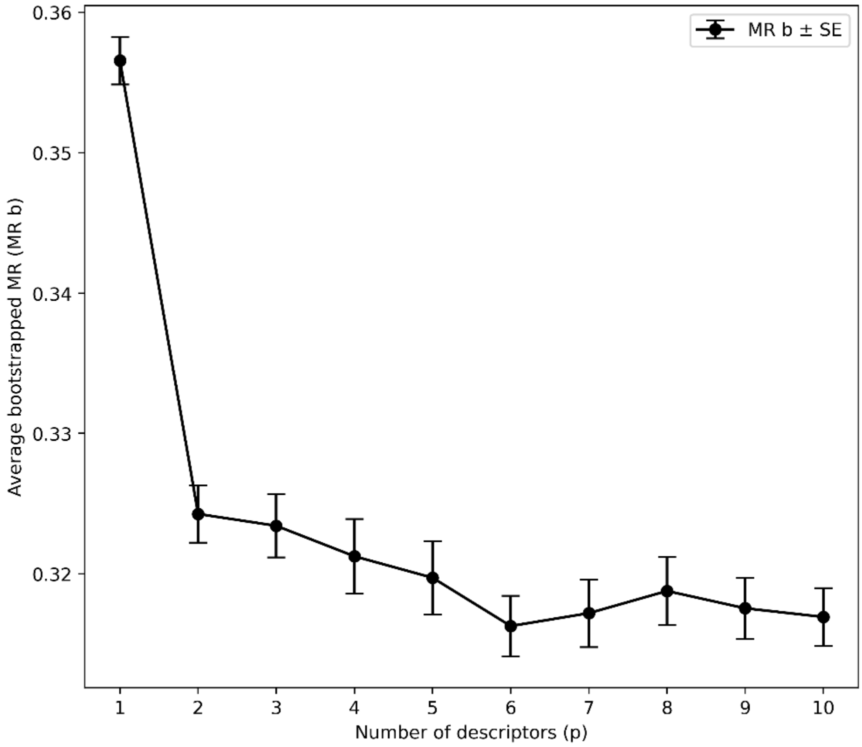

p	MR b	SE
1	0.356558933	0.001698796
2	0.324262971	0.002024297
3	0.32342188	0.00225317
4	0.32125024	0.002668237
5	0.319721646	0.002624858
6	0.316283942	0.002166223
7	0.317199614	0.002406126
8	0.318780161	0.002431088
9	0.317547372	0.002167543
10	0.316931758	0.002074926

Figure S1. Bootstrap analysis of the LDA-QSARs.

Linear scoring equations of the split LDA-QSAR

$$\boldsymbol{Class\ A\ score} = -49 + 28 \times \boldsymbol{GATS3e} + 79 \times \boldsymbol{ATSC6p} + 18 \times \boldsymbol{GATS8m} + 48 \times \boldsymbol{MIC2} + log\,(0.61)$$

(Equation S1)

$$\boldsymbol{Class\ I\ score} = -41 + 21 \times \boldsymbol{GATS3e} + 75 \times \boldsymbol{ATSC6p} + 14 \times \boldsymbol{GATS8m} + 42 \times \boldsymbol{MIC2} + log\,(0.39)$$

(Equation S2)

Where

A: active compounds;

I: weak/not active compounds.

Equation of the split MLR-QSAR

$$\boldsymbol{log\ RP} = -2.3\ (\pm 1.8) + 1.5\ (\pm 0.32) \times \boldsymbol{piPC5} - 3.1\ (\pm 0.92) \times \boldsymbol{GGI9} - 11\ (\pm 3.6) \times \boldsymbol{AATSC0e}$$

(Equation S3)

Where

$$RP = \frac{EC50_{T4}}{EC50_{COMPOUND}}$$

ROC curves of the split LDA-QSAR calculated for the training and test set, and for the full LDA-QSAR

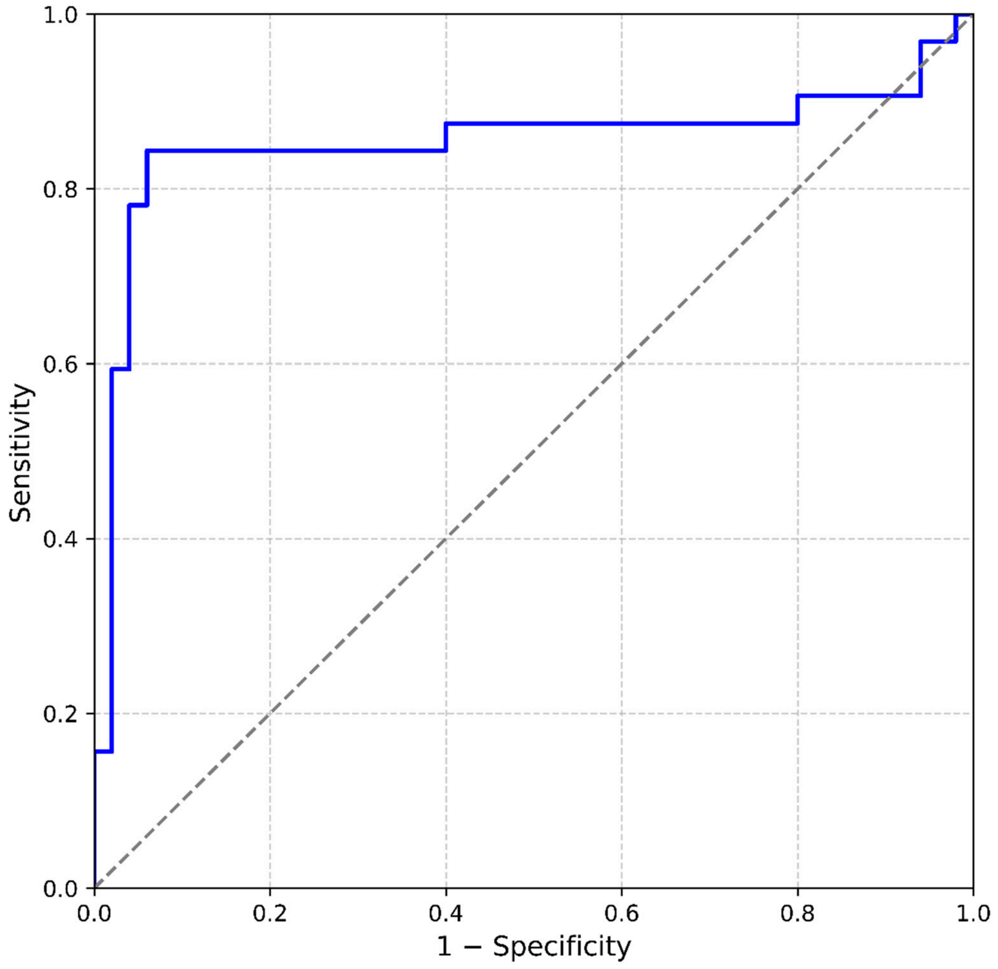

Figure S2. ROC curve of the split LDA-QSAR calculated for the training set.

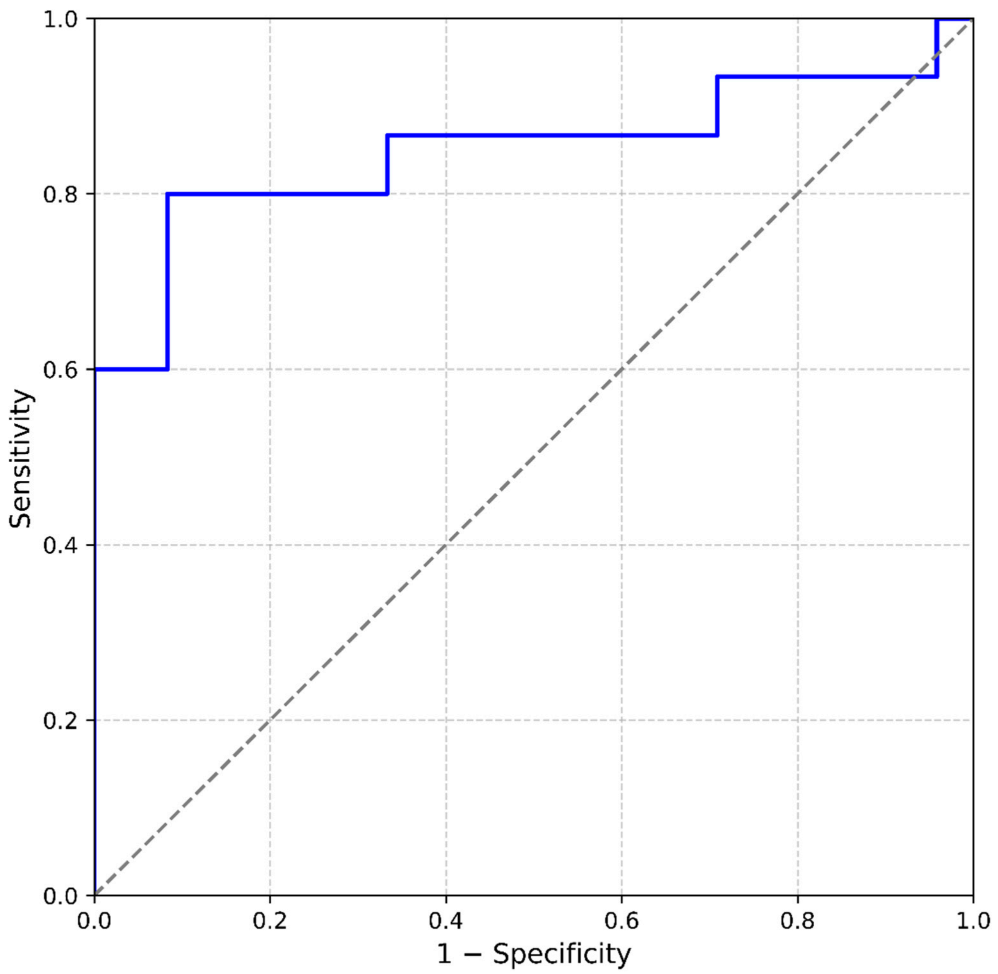

Figure S3. ROC curve of the split LDA-QSAR calculated for the test set.

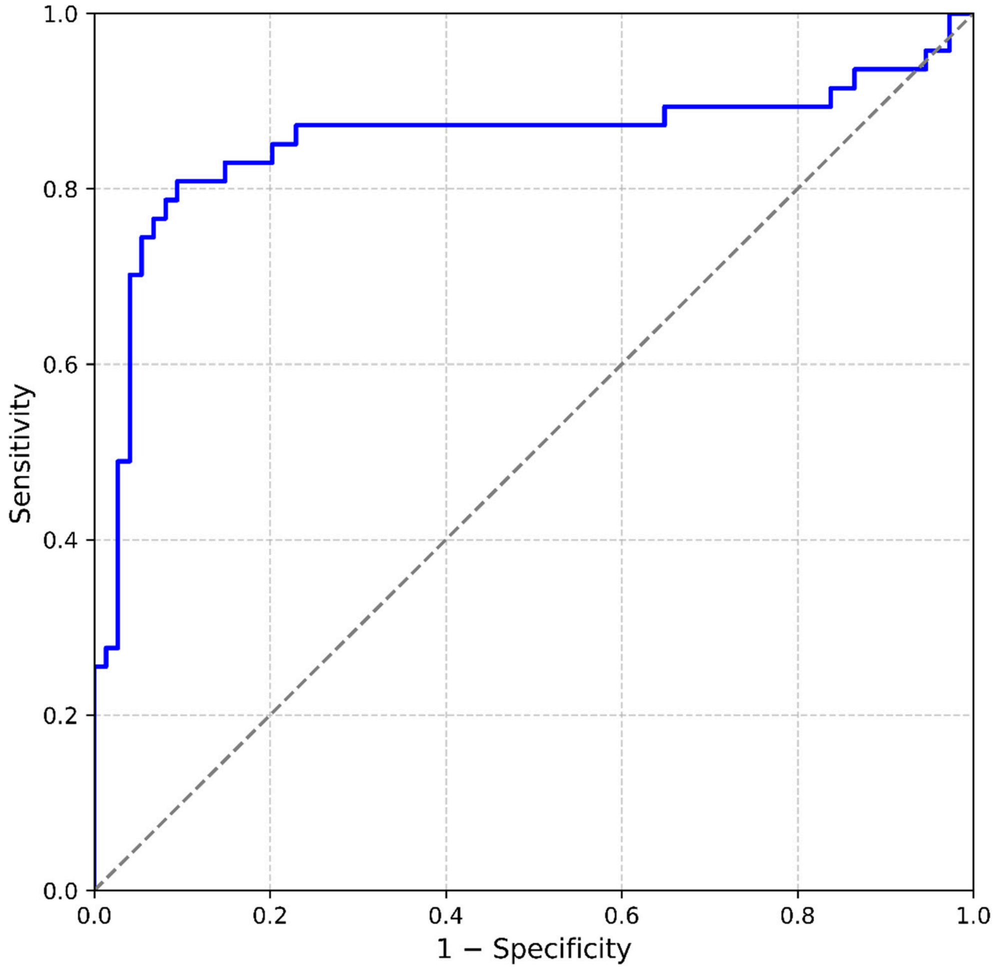

Figure S4. ROC curve of the full LDA-QSAR.

<u>Applicability domain plots (event A) of the LDA-QSAR (split and full)</u>

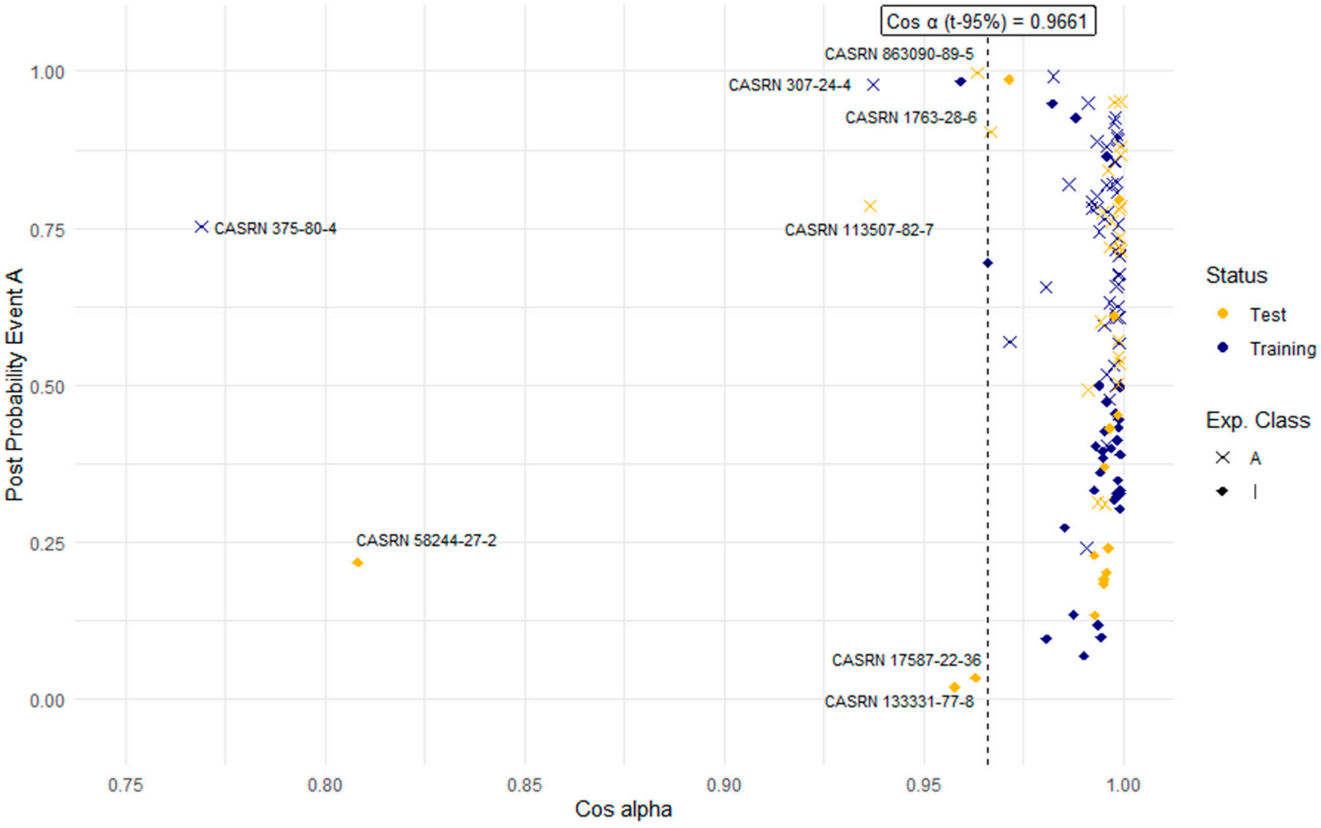

Figure S5. Applicability domain (AD) plot for the event A of the split LDA-QSAR.

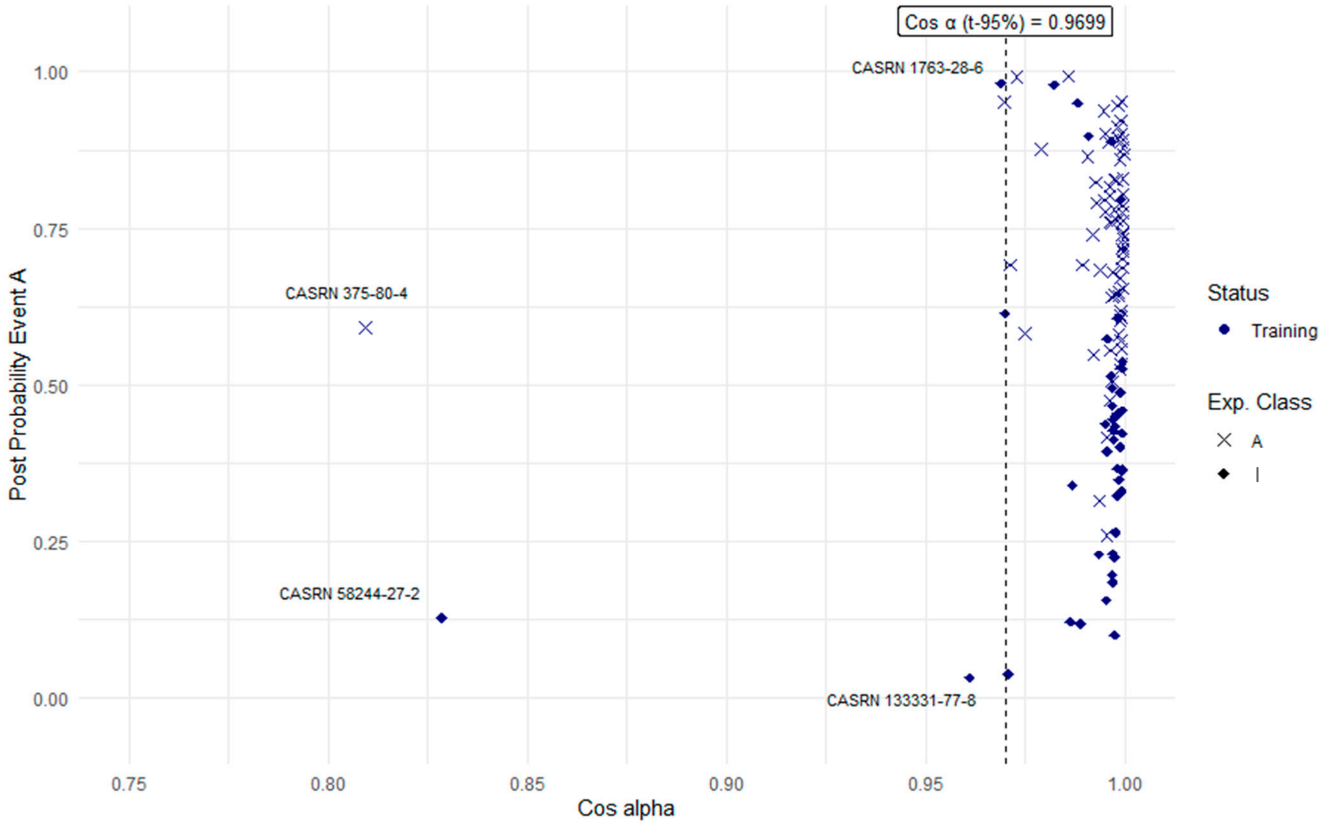

Figure S6. Applicability domain (AD) plot for the event A of the full LDA-QSAR.

<u>Bootstrap analysis: MLR-QSAR</u>

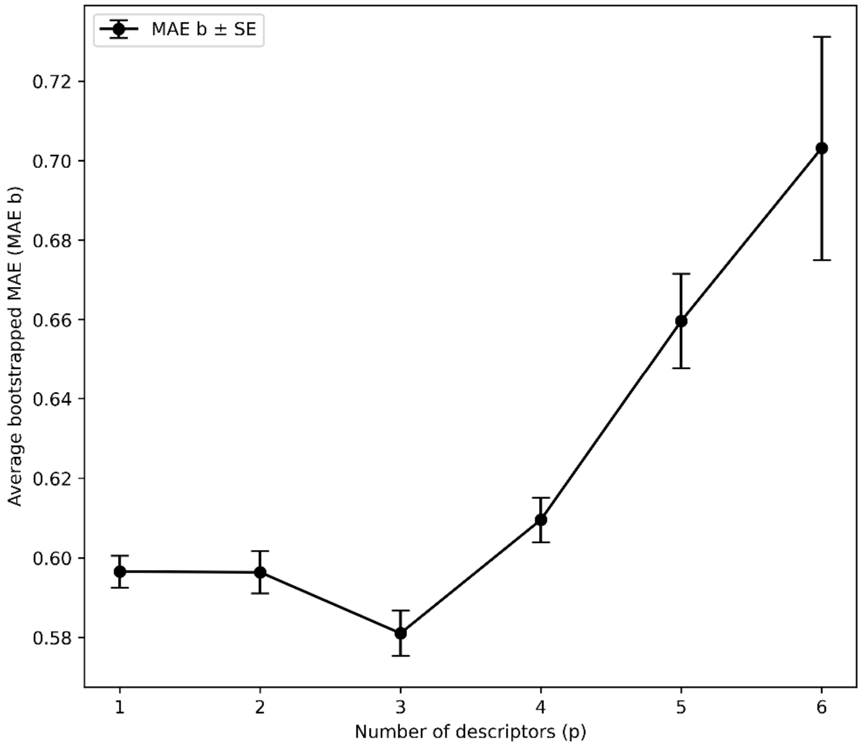

p	MAE b	SE
1	0.596557546	0.003988574
2	0.596406372	0.005319309
3	0.581063172	0.005699695
4	0.60958505	0.005612826
5	0.659684659	0.011911953
6	0.703180299	0.028098716

Figure S7. Bootstrap analysis of the MLR-QSARs.

Regression diagnostic plots of the MLR-QSAR: scatterplot of experimental vs predicted Log RP values (split and full)

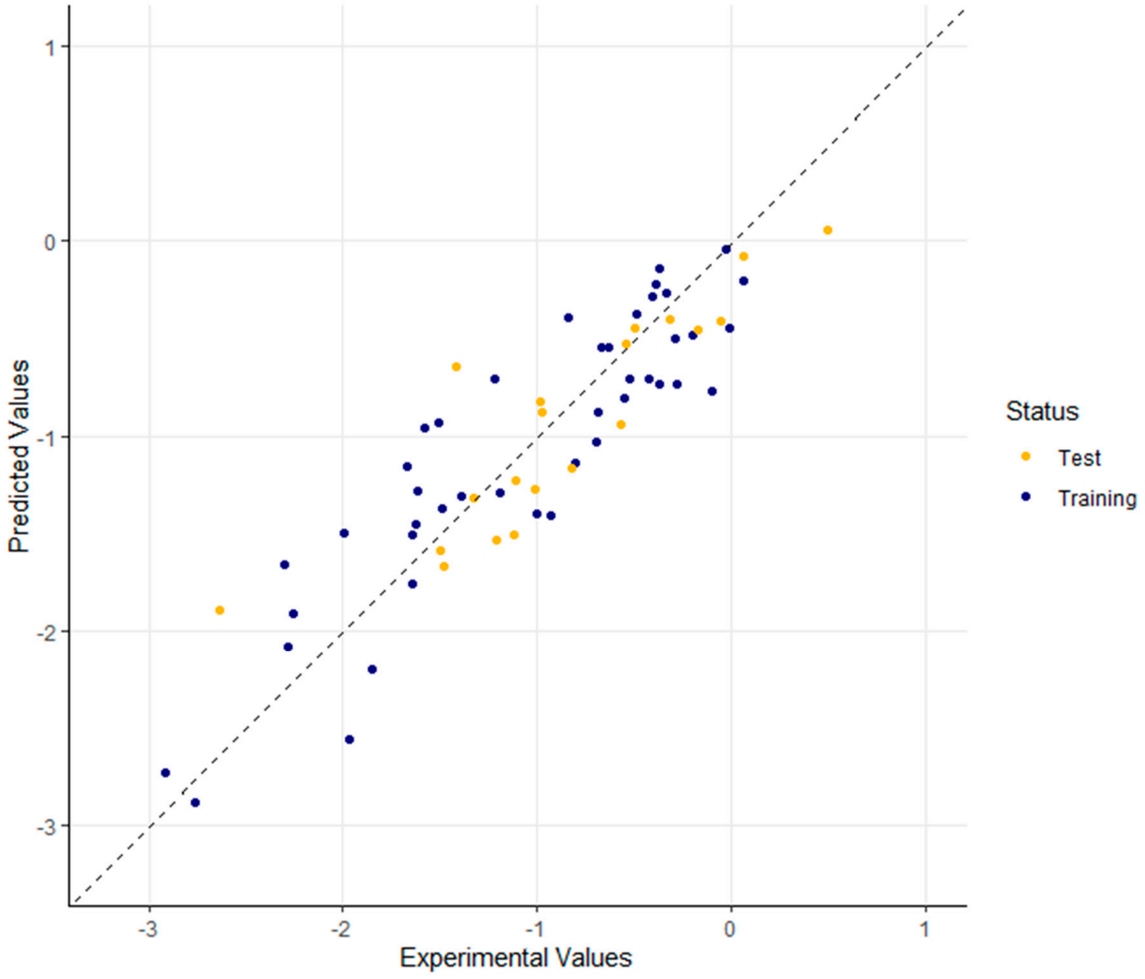

Figure S8. Plot of experimental vs predicted Log RP values of the split MLR-QSAR model.

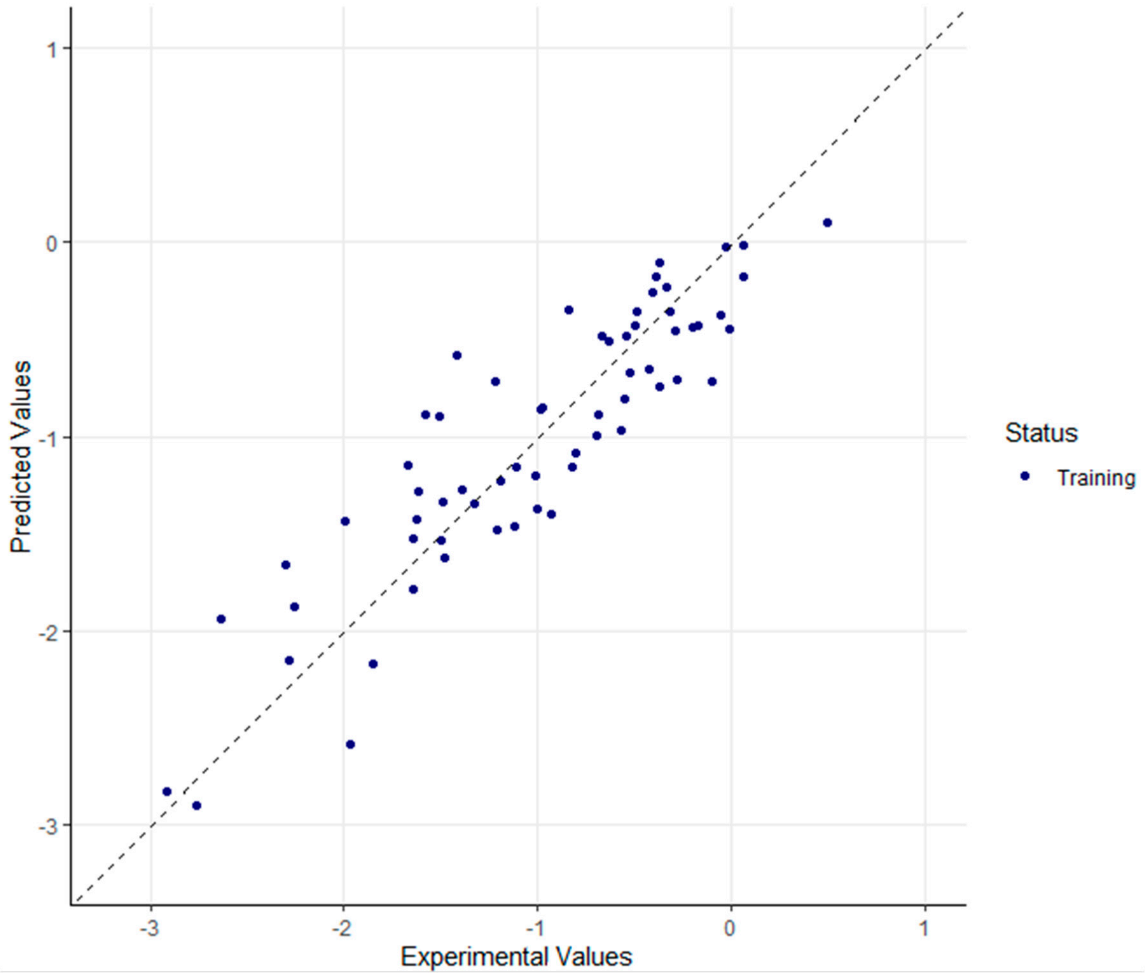

Figure S9. Plot of experimental vs predicted Log RP values of the full MLR-QSAR model.

Regression diagnostic plots of the MLR-QSAR: Residuals plot (split and full)

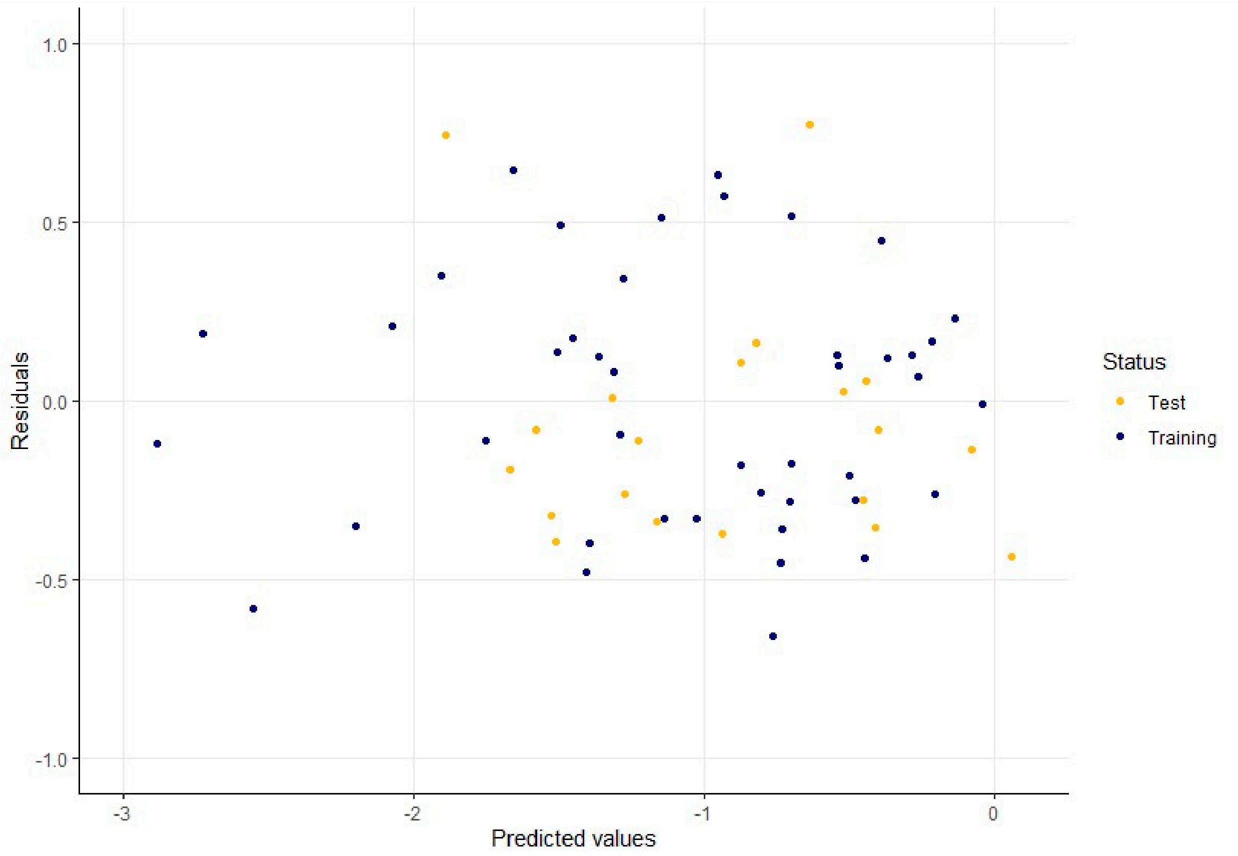

Figure S10. Residuals plot of the split MLR-QSAR model.

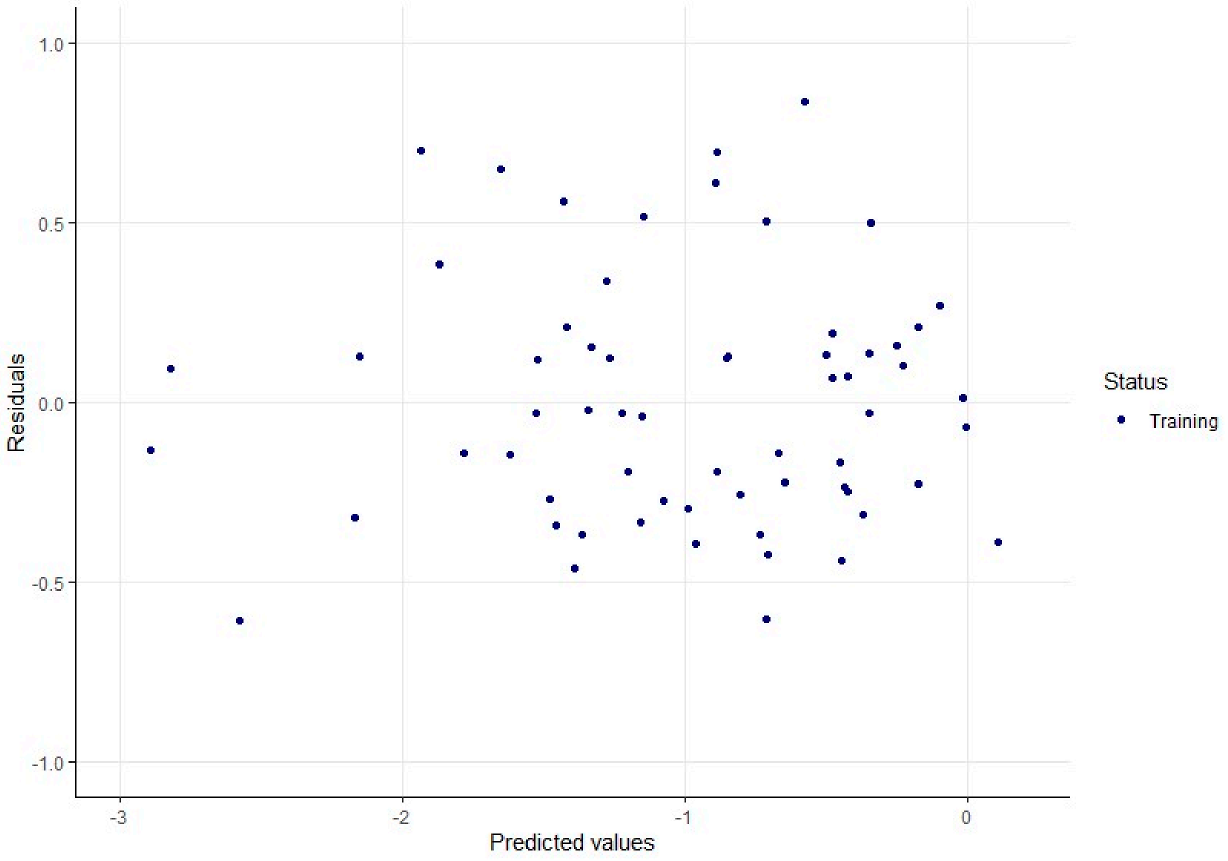

Figure S11. Residuals plot of the full MLR-QSAR model.

<u>Regression diagnostic plots of the MLR-QSAR: Williams plots (split and full)</u>

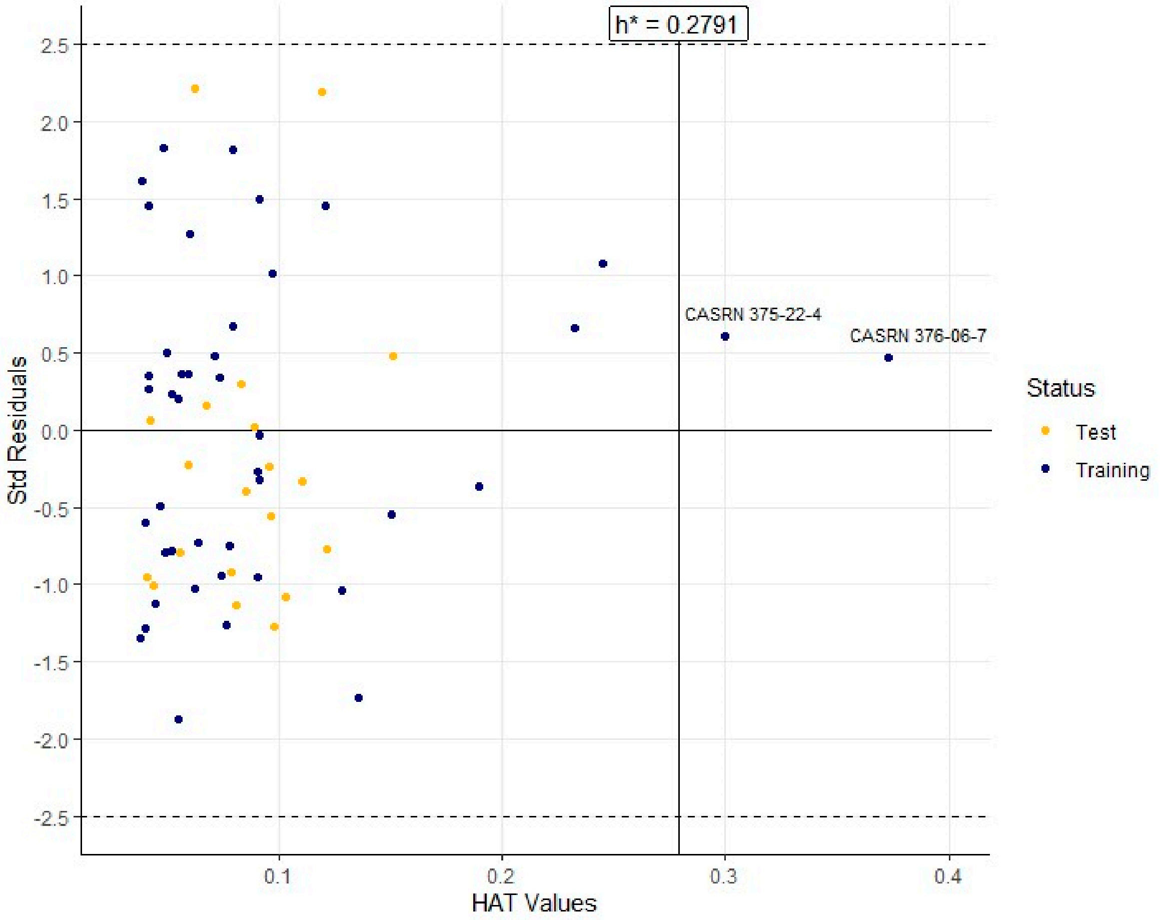

Figure S12. Williams plot of the split MLR-QSAR model.

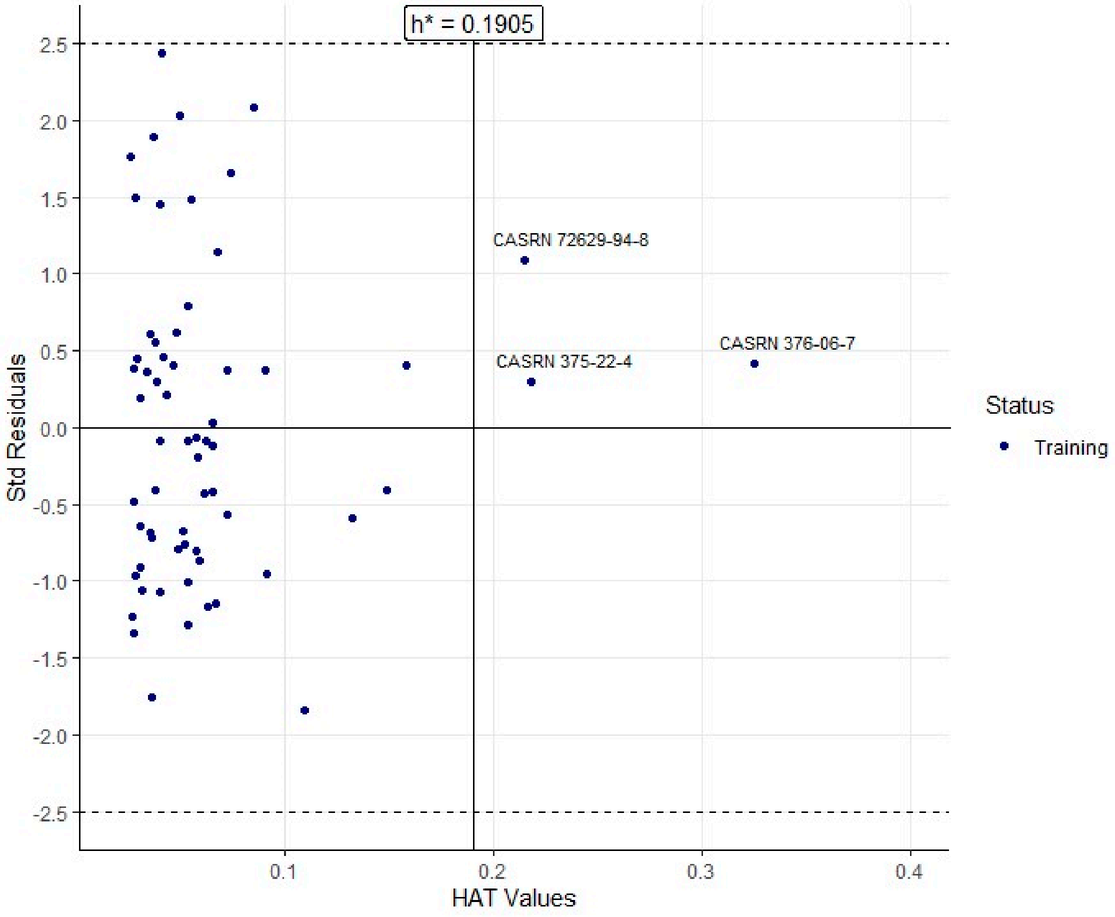

Figure S13. Williams plot of the full MLR-QSAR model.

Summary table of the selected molecular descriptors in the LDA-QSAR

Table S16. The molecular descriptors selected in Equation S1, Equation S2, Equation 1, and Equation 2, with their respective descriptions.

Molecular Descriptors	Description	Definition
GATS3e	Geary autocorrelation—lag 3/weighted by Sanderson electronegativities	$GATS_k = \frac{\frac{1}{2\Delta_k}\sum_{i=1}^{A}\sum_{j=1}^{A}(w_i - w_j)^2 \cdot \delta(d_{ij};k)}{\frac{1}{(A-1)}\sum_{i=1}^{A}(w_i - \bar{w})^2}$
ATSC6p	Centered Broto–Moreau autocorrelation—lag 6/weighted by polarizabilities	$ATSC_k = \frac{1}{2} \cdot \sum_{i=1}^{A}\sum_{j=1}^{A}(w_i - \bar{w})(w_j - \bar{w}) \cdot \delta(d_{ij};k)$
GATS8m	Geary autocorrelation—lag 8/weighted by mass	$GATS_k = \frac{\frac{1}{2\Delta_k}\sum_{i=1}^{A}\sum_{j=1}^{A}(w_i - w_j)^2 \cdot \delta(d_{ij};k)}{\frac{1}{(A-1)}\sum_{i=1}^{A}(w_i - \bar{w})^2}$
MIC2	Modified information content index (neighborhood symmetry of 2-order)	$MIC_m = -\sum_{g=1}^{G} m_g \cdot (p_g \log_2 p_g)$

Where

A: number of atoms in a molecule; w: atomic property; k: lag; d_{ij}: topological distance between *i-th* atom and *j-th* atom; $\delta(d_{ij};k)$: Kronecker delta function; $\bar{w}$: average of the atomic property on the molecule; Δ_k: sum number of vertex pairs at distance k; m: order of neighborhood information

content; G: number of equivalence classes; m_g: atomic mass of all the equivalent atoms in the g-th class; p_g: probability to select a vertex of class g.

Summary table of the selected molecular descriptors in the MLR-QSAR and correlation matrices (training set and full dataset)

Table S17. The molecular descriptors selected in Equation S3 and Equation 3, with their respective descriptions.

Molecular Descriptors	Description	Definition
piPC5	Conventional bond order ID number of order 5 (ln(1+x))	$piPCk = \ln(1 + \sum_{k_{p_{ij}}} w_{ij})$
GGI9	Topological charge index of order 9	$G_k = \frac{1}{2} \cdot \sum_{i=1}^{A} \sum_{J=1}^{A} \lvert CT_{ij} \rvert \cdot \delta(d_{ij}; k)$
AATSC0e	Average centered Broto–Moreau autocorrelation—lag 0/weighted by Sanderson electronegativities	$\overline{ATS}_0 = \frac{1}{\Delta_k} \cdot \sum_{i=1}^{A} (w_i - \overline{w})^2$

Where

$k_{p_{ij}}$ is a path length k from vertex i to vertex j; w_{ij} is the path weight resulting from the products of bond orders in the path; A is the number of atoms in a molecule; CT_{ij} is the charge term matrix for each pair of vertices v_i and v_j; $\delta(d_{ij}; k)$ is the Kronecker delta function; Δ_k is the sum number of vertex pairs at distance k; w is the atomic property; k is the lag; d_{ij} is the topological distance between the *i-th* atom and the *j-th* atom; $\overline{w}$ is the average of the atomic property on the molecule.

Table S18. Correlation matrix of the selected molecular descriptors in the MLR-QSAR for the training set of the split model.

	piPC5	GGI9	AATSC0e
piPC5	1	0.69	− 0.20
GGI9	0.69	1	− 0.14
AATSC0e	− 0.20	− 0.14	1

Table S19. Correlation matrix of the selected molecular descriptors in the MLR-QSAR for the full dataset.

	piPC5	GGI9	AATSC0e
piPC5	1	0.68	− 0.04
GGI9	0.68	1	− 0.03
AATSC0e	− 0.04	− 0.03	1

Summary table of the predicted PFAS with data from *in vitro* experimental studies.

Table S20. List of PFAS, experimental or predicted activity/inactivity, and literature references. *In the original reference, the compound was indicated as 6:2 Cl-PFAES, while no CAS No was reported. ** In the original reference, the compound was indicated as 2H-Perfluoro-2-octenoic acid (6:2), or FTUA (6:2), while the CAS No was reported as not available (n.a.).

CASRN	Outcome	Reference
13252-13-6	Active	This study
	Active	[2]
	Active	[3]
	Active	[4]
1546-95-8	Active	This study
	Active	[4]
	Active	[5]
30334-69-1	Active	This study
	Active	[3]
	Active	[4]
52299-26-0	Active	This study
	Active	[6]
2706-91-4	Active	This study
	Active	[4]
172155-07-6	Active	This study
	Active	[4]
919005-14-4	Active	This study
	Active	[4]

756426-58-1	Active	This study
	Active	[7]*
70887-88-6	Active	This study
	Active	[5]**
335-77-3	Active	This study
	Active	[3]
	Inactive	[4]
	Inactive	[5]
57678-01-0	Active	This study
	Inactive	[6]
40143-76-8	Active	This study
	Inactive	[6]

Analysis of the predictions of the QSARs within structural subcategories

Analysis of the predictions of the LDA-QSAR within structural subcategories:

“**Perfluoroalkyl phosphate compounds**” was the structural category with the highest percentage of active predictions. However, this result was limited due to the small size of the category (eight PFAS, distributed across two structural subcategories), which was the least represented one in the OECD List after the data curation procedure.

“**Other perfluoroalkyl acids (PFAA) precursors and related compounds—perfluoroalkyl ones**”, which included 161 unique PFAS distributed across ten structural subcategories, was the second most represented structural category in terms of the percentage of active predictions. Six out of the ten structural subcategories were predominantly characterized by active predictions (from 83.3 % to 100 %), where “perfluoroalkanes and aromatics” and “perfluoroalkanes and derivatives” were the two subcategories of major concern, considering the numbers of active compounds out of their total sums (51 out of 52, and 40 out of 41, respectively). “Perfluoroalkyl halides (other than iodides)”, “perfluoroalkyl amines”, “perfluoroalkyl ketons”, and “perfluoroalkyl cyanides” remained potential causes of concern since almost all the PFAS belonging to these subcategories were predicted as active; however, their small size should be taken into account (16, 12, 11, and 4 PFAS, respectively). The remaining four subcategories showed a more balanced distribution between active and weak/not active PFAS (from 50 % to 70 % of actives). However, also in this case, it is important to take into account the small sizes of these subcategories, which included from 2 to 10 PFAS.

“**Per- and polyfluoroalkyl ether-based compounds**” was the third structural category of major concern, since 95 out of the 119 PFAS included in this category (distributed across eighteen structural subcategories, whose size ranged from 1 to 22 PFAS) were predicted as active. Fourteen out of these subcategories had active prediction percentages higher than 70.0 %, with “perfluoroalkyl ethers/alkanes + aromatics—monoethers” and “perfluoroalkyl ether carboxylic acids (PFECAs), salts and esters—diethers” being particularly noteworthy due to their size (15 and 18 PFAS, respectively) and percentage of active predictions (100% and 83.3 %, respectively). The remaining four subcategories, i.e., “per- and polyfluoroalkyl ether + telomer-based substances—triethers”, “PFECAs, salts and esters—monoethers”, “PFECA-related substances—monoethers”, and “PFECA-related substances—diethers” were characterized by a higher proportion of weak/not active predictions, which was 100 % for the latter one.

Almost half of the PFAS belonging to the structural categories “**perfluoroalkane sulfonyl compounds**”, “**perfluoroalkyl carbonyl compounds**”, and “**fluorotelomer-related compounds**” had a great ability to bind to hTTR. “**Perfluoroalkane sulfonyl compounds**”, which included 124 PFAS across nine subcategories, showed a variable distribution of active and weak/not active

predictions, which was not consistent within the structure category. While some subcategories were dominated by positive predictions, others were primarily composed of negative ones. This was particularly evident when analyzing the three largest subcategories, which included the 70.2 % of the PFAS belonging to the whole category, i.e., "perfluoroalkane sulfonyl-based nonpolymers", "perfluoroalkane sulfonic acids (PFSAs), their salts and esters", and "perfluoroalkane sulfonyl amides/amido ethanols (xFASA/Es) and other alcohols" (composed of 44, 26, and 17 PFAS, respectively). In the first subcategory, the number of active predictions (23) was similar to the weak/not active ones (21); in the second subcategory, the number of active predictions (24) exceeded the number of weak/not active predictions (2); in the third subcategory, the number of weak/not active predictions (13) far exceeded the number of active predictions (4).

"**Perfluoroalkane carbonyl compounds**" was composed of 156 PFAS across nine subcategories. As was seen for "**perfluoroalkane sulfonyl compounds**", the ratio of active and weak/not active predictions for each subcategory was not consistent within the structural category. Again, this inconsistency was made particularly evident by analyzing the four largest subcategories, which included the 94.2 % of the PFAS belonging to the whole category (i.e., "**perfluoroalkane carbonyl compounds**"). "Perfluoroalkyl carboxylic acids (PFCAs), their salts and esters" was composed of 61 PFAS, 80.3 % of which were predicted as active; meanwhile, all the PFAS categorized as "perfluoroalkyl carbonyl halides" (18) were predicted as active. On the contrary, "other perfluoroalkyl carbonyl-based nonpolymers" and "perfluoroalkyl carbonyl amides/amido ethanols and other alcohols" (characterized by 50 and 18 PFAS, respectively) were the two subcategories predominantly composed of weak/not active predictions (92.0 % and 94.4 %, respectively).

The third structural category with an almost equal ratio of active and weak/not active predictions was represented by "**fluorotelomer-related compounds**", which was the largest one in terms of the number of PFAS (436) and the subcategories (24) that fell within the AD of the model. Also in this case, the ratio of active and weak/not active predictions for each subcategory was not consistent within the structural category. This scenario was clarified by considering the nine most represented subcategories (each one characterized by more than ten compounds), which included the 87.4 % of the PFAS belonging to the whole category. Three subcategories, i.e., "perfluoroalkyl iodides (PFAIs)", "n:2 fluorotelomer alcohol, phosphate esters (PAPs)", and "n:2 fluorotelomer-based (meth)acrylate" (composed of 26, 20, and 11 PFAS, respectively) were of greatest concern, since they were characterized by a higher percentage of active predictions (100 %, 95 %, and 81.8 %, respectively). Another three subcategories, i.e., "n:2 fluorotelomer iodides (n:2 FTIs)", "n:1 fluorotelomer alcohols", and "n:2 fluorotelomer alcohols (n:2 FTOHs)/thiols" (composed of 51, 15, and 12 PFAS, respectively) were characterized by a percentage of active predictions nearly similar to the weak/not active ones (58.8 %, 46.7 %, and 41.7 %, respectively). Contrarily, the remaining three large subcategories, i.e., "n:2 fluorotelomer-based non-polymers", "n:1 fluorotelomer-based non-polymers", and "n:2 FT-thiol derivatives" (composed of 177, 57, and 12 PFAS, respectively) showed a higher percentage of weak/not active predictions (62.1 %, 71.9 %, and 75.0 %, respectively). It is important to highlight that, even though the subcategory "n:2 fluorotelomer-based non-polymers"

was characterized by a lower percentage of active predictions (37.9 %), it remains of relative concern due to its large size (177 PFAS).

Finally, "**other PFAA precursors or related compounds—semifluorinated**" (composed of 279 unique PFAS distributed across nine structural subcategories) was the only structural category predominantly characterized by weak/not active predictions. Two subcategories were of notable relevance, i.e., "side-chain fluorinated aromatics", and "semifluorinated alkanes (SFAs) and derivatives", due to their size (147 and 72 PFAS, respectively) and the percentage of weak/not active predictions (81 % and 88 %, respectively). Five additional subcategories showed a percentage of weak/not active predictions higher than active ones (from 63.6 % to 100 %), even though their small size should be taken into account (from 1 to 22 PFAS). The remaining two subcategories, i.e., "hydrofluoroolefins (HFOs)", and "hydrofluoroethers (HFEs) and derivatives", may be of concern due to being characterized by a higher percentage of active predictions (90.9 % and 66.7 %, respectively). Again, their small size should be considered (11 and 3 PFAS, respectively).

Analysis of the predictions of the MLR-QSAR within structural subcategories:

"**Other PFAA precursors and related compounds—perfluoroalkyl ones**" was the structural category of major concern, since 74 PFAS (out of 81, distributed across ten structural subcategories) were predicted as strong hTTR binders. "Perfluoroalkyl halides (other than iodides)", "perfluoroalkenes and derivatives", "perfluoroalkanes and aromatics", and "perfluoroalkyl ketons", which included the 77.8 % of the PFAS belonging to the whole category (i.e., "**other PFAA precursors and related compounds—perfluoroalkyl ones**"), were the four structural subcategories of most notable relevance due to their size (12, 26, 17, and 8 PFAS, respectively) and the percentage of strong hTTR binders (100 %, 88.5 %, 88.2 %, and 75 %, respectively). PFAS included in the remaining six subcategories were all predicted as strong hTTR binders, even though the small size of these subcategories should be taken into account (from one to five PFAS).

"**Perfluoroalkyl carbonyl compounds**" was the second most represented structural category in terms of strong hTTR binders (50 out of 56 PFAS). In this case, among the six structural subcategories across which these PFAS were distributed, "perfluoroalkyl carboxylic acids (PFCAs), their salts and esters" and "perfluoroalkyl carbonyl halides" were the two of most relevance due to their size (35 and 13 PFAS, respectively) and the percentage of strong hTTR binders (94.3 % and 84.6 %, respectively). PFAS included in the remaining four subcategories were predicted as strong hTTR binders with a percentage that ranged from 50 % to 100 %, but their small size (two PFAS for each one) should still be taken into account.

“**Per- and polyfluoroalkyl ether-based compounds**”, “**perfluoroalkane sulfonyl compounds**”, and “**perfluoroalkyl phosphate compounds**” were structural categories characterized by similar great percentages of strong hTTR binders. Regarding “**per- and polyfluoroalkyl ether-based compounds**” (composed of 69 PFAS distributed across fifteen subcategories), the PFAS belonging to subcategories such as “PFECAs, salts and esters—monoethers”, “PFECAs, salts and esters—diethers”, and “perfluoroalkyl ethers/alkanes + aromatics—monoethers” were of most concern, due to their size (13, 12, and 9 PFAS, respectively) and the percentage of strong hTTR binders (76.9 %, 91.7 %, and 88.9 %, respectively). The number of PFAS and the percentage of strong hTTR binders in the remaining twelve subcategories ranged from one to five and from 0 % to 100 %, respectively.

Regarding “**perfluoroalkane sulfonyl compounds**” (composed of 44 PFAS distributed across seven subcategories), the PFAS belonging to the subcategories “perfluoroalkane sulfonic acids (PFSAs), their salts and esters” and “perfluoroalkane sulfonyl halides” were of most concern due to their numbers (19 and 8, respectively) and the percentage of strong hTTR binders (84.2 % and 100 %, respectively). The number of PFAS and the percentage of strong hTTR binders in the remaining five subcategories ranged from two to six and from 50.0 % to 83.3 %, respectively.

Results obtained for “**perfluoroalkyl phosphate compounds**”, even though they may be of concern, were limited due to the small size of the category (a total of five PFAS distributed across two subcategories).

“**Fluorotelomer-related compounds**” was still the largest structural category, since it was composed of 147 PFAS distributed across nineteen subcategories. While some subcategories were dominated by strong hTTR binders, others were primarily characterized by PFAS of lower hTTR binding strength. This was made particularly evident by considering their distribution across the four largest subcategories, which included almost 60 % of the PFAS belonging to “**fluorotelomer-related compounds**”, i.e., “n:2 fluorotelomer-based non-polymers”, “n:2 fluorotelomer iodides (n:2 FTIs)”, “perfluoroalkyl iodides (PFAIs)”, and “n:1 fluorotelomer-based non-polymers” (numbers of PFAS: 45, 17, 14, and 12, respectively; percentages of strong hTTR binders: 75.6 %, 23.5 %, 85.7 %, and 41.7 %, respectively; percentages of non-strong hTTR binders: 24.4 %, 76.5 %, 14.3 %, and 58.3 %, respectively). The numbers of PFAS and the percentages of strong hTTR binders in the remaining fifteen subcategories ranged from one to eight and from 0 % to 100 %, respectively.

The last structural category, i.e., “**other PFAA precursors or related compounds—semifluorinated**”, was the one of least concern, since it included the lowest percentage of strong hTTR binders. However, the small size of this category should be taken into consideration (a total of 16 PFAS distributed across six categories). Three subcategories, i.e., “hydrofluoroolefins (HFOs)” (seven PFAS), “semi-fluorinated ketons” (two PFAS), and “side-chain fluorinated aromatics” (one compound), were of relative concern, compared to the others, due to the percentage of strong hTTR binders (57.1 %, 50.0 %, and 100 %, respectively). The remaining three subcategories were not characterized by strong hTTR binders.

References

1. Rücker, C.; Rücker, G.; Meringer, M. Y-Randomization and Its Variants in QSPR/QSAR. *J. Chem. Inf. Model.* **2007**, *47*, 2345–2357, https://doi.org/10.1021/ci700157b.

2. Carlier, M.P.; Cenijn, P.H.; Baygildiev, T.; Irwan, J.; Escher, S.E.; van Duursen, M.B.M.; Hamers, T. Profiling the Endocrine-Disrupting Properties of Triazines, Triazoles, and Short-Chain PFAS. *Toxicol. Sci.* **2024**, *202*, 250-264, https://doi.org/10.1093/toxsci/kfae131.
3. Langberg, H.A.; Choyke, S.; Hale, S.E.; Koekkoek, J.; Cenijn, P.H.; Lamoree, M.H.; Rundberget, T.; Jartun, M.; Breedveld, G.D.; Jenssen, B.M.; Higgins, C.P.; Hamers, T. Effect-Directed Analysis Based on Transthyretin Binding Activity of Per- and Polyfluoroalkyl Substances in a Contaminated Sediment Extract. *Environ. Toxicol. Chem.* **2024**, *43*, 245–258, https://doi.org/10.1002/etc.5777.
4. Sosnowska, A.; Mudlaff, M.; Mombelli, E.; Behnisch, P.; Zdybel, S.; Besselink, H.; Kuckelkorn, J.; Bulawska, N.; Kepka, K.; Kowalska, D.; Brouwer, A.; Puzyn, T. Identification of New PFAS for Severe Interference with Thyroid Hormone Transport: A Combined in Vitro/Silico Approach. *J. Hazard Mater.* 2025, *491*, 137949, https://doi.org/10.1016/j.jhazmat.2025.137949.
5. Weiss, J.M.; Andersson, P.L.; Lamoree, M.H.; Leonards, P.E.G.; van Leeuwen, S.P.J.; Hamers, T. Competitive Binding of Poly- and Perfluorinated Compounds to the Thyroid Hormone Transport Protein Transthyretin. *Toxicol. Sci.* **2009**, *109*, 206–216, https://doi.org/10.1093/toxsci/kfp055.
6. Zhang, J.; Kamstra, J.H.; Ghorbanzadeh, M.; Weiss, J.M.; Hamers, T.; Andersson, P.L. In Silico Approach To Identify Potential Thyroid Hormone Disruptors among Currently Known Dust Contaminants and Their Metabolites. *Environ. Sci. Technol.* **2015**, *49*, 10099–10107, https://doi.org/10.1021/acs.est.5b01742.
7. Xin, Y.; Ren, X.-M.; Ruan, T.; Li, C.-H.; Guo, L.-H.; Jiang, G. Chlorinated Polyfluoroalkylether Sulfonates Exhibit Similar Binding Potency and Activity to Thyroid Hormone Transport Proteins and Nuclear Receptors as Perfluorooctanesulfonate. *Environ. Sci. Technol.* **2018**, *52*, 9412–9418, https://doi.org/10.1021/acs.est.8b01494.
